# Advances in Antiviral Delivery Systems and Chitosan-Based Polymeric and Nanoparticulate Antivirals and Antiviral Carriers

**DOI:** 10.3390/v15030647

**Published:** 2023-02-28

**Authors:** Dominika Žigrayová, Veronika Mikušová, Peter Mikuš

**Affiliations:** 1 Department of Galenic Pharmacy, Faculty of Pharmacy, Comenius University Bratislava, Odbojárov 10, 83232 Bratislava, Slovakia; 2 Department of Pharmaceutical Analysis and Nuclear Pharmacy, Faculty of Pharmacy, Comenius University Bratislava, Odbojárov 10, 83232 Bratislava, Slovakia; 3 Toxicological and Antidoping Center, Faculty of Pharmacy, Comenius University Bratislava, Odbojárov 10, 83232 Bratislava, Slovakia

**Keywords:** chitosan, nanoparticles, antivirotic, drug delivery, chitosan derivatives, chitosan nanocomposites

## Abstract

Current antiviral therapy research is focused on developing dosage forms that enable highly effective drug delivery, providing a selective effect in the organism, lower risk of adverse effects, a lower dose of active pharmaceutical ingredients, and minimal toxicity. In this article, antiviral drugs and the mechanisms of their action are summarized at the beginning as a prerequisite background to develop relevant drug delivery/carrier systems for them, classified and briefly discussed subsequently. Many of the recent studies aim at different types of synthetic, semisynthetic, and natural polymers serving as a favorable matrix for the antiviral drug carrier. Besides a wider view of different antiviral delivery systems, this review focuses on advances in antiviral drug delivery systems based on chitosan (CS) and derivatized CS carriers. CS and its derivatives are evaluated concerning methods of their preparation, their basic characteristics and properties, approaches to the incorporation of an antiviral drug in the CS polymer as well as CS nanoparticulate systems, and their recent biomedical applications in the context of actual antiviral therapy. The degree of development (i.e., research study, in vitro/ex vivo/in vivo preclinical testing), as well as benefits and limitations of CS polymer and CS nanoparticulate drug delivery systems, are reported for particular viral diseases and corresponding antivirotics.

## 1. Introduction

Among all the disease-causing agents in humans, viruses are the most notorious, active, and important [1]. Infections by viruses in humans cause millions of deaths around the globe and are accountable for human diseases such as HIV/AIDS, hepatitis, influenza, herpes simplex, common cold, etc. The upsurge in chronic viral infections such as HIV, HCV, HBV, etc., and the emergence of new viruses such as severe acute respiratory syndrome coronavirus (SARS-CoV, causing SARS) and severe acute respiratory syndrome coronavirus 2 (SARS-CoV-2, causing COVID-19), emphasize the growing need for novel strategies to develop antiviral agents. The majority of antiviral drugs are small molecules with diverse roles in clinical use. The large molecules include proteins (interferons, monoclonal antibodies), peptides, and oligonucleotides. Most of these antiviral drugs target the virus’s cellular machinery, while very few of them target the host cells/cellular mechanisms [2]. The advances in antiviral drug discoveries were discussed in the review of Saxena et al. [1]. Menendez-Arias and Delgado [3] focused their very recent review on the latest updates in antiretroviral therapy.

Clinical use of the currently available antiviral drugs is limited by various factors such as toxic side effects and possible viral latency [1]. There are also some general limitations inherent in antiviral chemotherapy. First, the more selective the antiviral drug, the narrower its antiviral activity spectrum. Second, since antiviral drugs target steps in virus replication, the latent phases characteristic of some viral (i.e., herpesviral) infections are not amenable to chemotherapy. This is particularly relevant for herpesvirus and retrovirus infections. Thus, eradication of latent virus infections is not feasible currently. Third, antiviral drug treatment should be started early, before irreversible tissue damage occurs. Such timely treatment is not possible without an early and accurate diagnosis, which is difficult for many viral infections (such as infections of the respiratory tract). Fourth, perhaps inevitably for a specific antimicrobial agent, there is the risk of the emergence of drug-resistant virus strains [1,4].

One of the major challenges associated with antiviral drugs is bioavailability, affected by a drug’s ability to be absorbed by the gastrointestinal tract. [5]. An approach oriented toward the development of novel drug delivery systems can be used to achieve efficacious therapy by improving the design, formulation, and delivery of existing antiviral agents. Innovative dosage forms help to decrease toxicity and dosage and may enhance the stability, specificity, and efficiency of antivirals [6]. Sharma et al. and Durai [6,7], in their review papers, discussed the different approaches to drug delivery of antiviral and antiretroviral drugs. Currently, among the most frequently studied systems for antiviral drug delivery are the following: modified-release forms such as depot tablets, multi-unit particulate system tablets [8], floating systems, implants [9], films, micro- and nanoemulsions, and drug carriers such as lipid nanoparticles (NPs) as liposomes [10] or ethosomes [11], solid lipid NPs [12], carbon-based NPs [13] and polymeric NPs [14].

NPs can be defined as submicron colloidal drug carrier systems ranging in size between 10 and 100 nm. Advanced nanoparticulate drugs are more suitable than conventional medicines in terms of site-specific targeting capabilities, sustained and controlled release, increased absorption rates and bioavailability, and improved stability of therapeutic agents. The NP size, hydrophobicity, modified surface, high surface-to-volume ratio, and surface charge are the essential factors that control the targeting capabilities, help in reducing the drug dose and frequency of administration, leading to reduced toxicity and side effects of drugs, and thus improve patient compliance [15,16]. Recently, the FDA published its guidance on “drug products, including biological products, that contain nanomaterials” [17], in doing so establishing the attributes of nanomaterials. The average particle size, particle size distribution (PSD), shape, morphology, surface charge, and concentration, among others, are mentioned as mandatory attributes to be determined [18].

Delshadi et al. [19] discussed, in their review, the ways to overcome the limitations of current antivirals by creating nanoparticulate drug delivery systems. In addition, Maus et al. [5] and Lembo et al. [20] discussed various types of NPs as effective carriers for antiviral drugs. Wang et al. [21] focused on the biosafe nanomaterials used for antiviral therapy and discussed the options for the design of antiviral drugs in the future.

Currently, polymers are being widely used for pharmaceutical applications. Polymeric nanosystems have unique physicochemical characteristics such as prolonged blood circulation time, reduced adverse effects, ability to protect therapeutic agents from degradation, and increased stability. They also increase bioavailability of drugs, can be easily chemically modified, provide controlled drug release, and have high efficacy [22]. One of the most frequently used polymers is CS due to its beneficial properties such as biodegradability, biocompatibility, the ability for wound healing, antimicrobial activity, and, most importantly, mucoadhesiveness, making it a good carrier material in drug delivery systems [23]. CS, being positively charged and engaged in interaction with the mucus membrane, is capable of opening the tight junctions between cells, thus promoting passage through the mucosal cells and enhancing drug permeation [24]. Although CS possesses many functional properties, it has also several limitations such as high hydrophilicity, low ductility, a high degree of swelling, and low thermal stability. A major limiting factor is its poor water solubility. CS is insoluble at a physiological pH (pH 7.4) and ineffective as an absorption enhancer, which interferes with its biomedical application. Improving the solubility of CS is a crucial factor for judicious use in a multitude of applications, which is achieved through chemical modification of CS. Chemical derivatives of CS have received increasing interest over the past decade due to their advocated chemical, biological, and functional advantages over unmodified CS based on their solubility, gelling properties, hydrophobic derivatives with amphiphilic character, capacity to harness self-assembling nanostructures and chemical conjugates with an assortment of bioactive and therapeutic molecules, improved biocompatibility, and enhanced properties for complexing biomolecules (e.g., DNA, RNA) [16]. Classification of the CS derivatives and their methods of preparation, along with the improvement of properties and applications, was offered in recent reviews by Mikušová and Mikuš [16] and Negm et al. [25]. Wang et al. [26] discussed CS derivatives and their applications in biomedicine and Patrulea et al. [27] in wound healing.

The present review paper informs about updates in the area of CS and its role in current pharmaceutical systems for antivirotics. Before discussing the strategies in the development of effective CS dosage forms (Section 4), various antiviral therapy approaches are described (including current antivirals and their mechanisms of action (Section 2), current antiviral delivery approaches, and dosage forms (Section 3)). Then, the article continues with a comprehensive review of CS and its derivatives relevant to antivirotics’ formulation, along with the current state of CS-based polymeric (Section 4.1) as well as NP (Section 4.2) systems proposed for antiviral therapy, either composed of CS derivatives possessing antiviral properties or CS NPs loaded with antivirotics. The advantages and limitations of various CS-based delivery systems for antivirotics are discussed, along with their potential for practical implementation.

## 2. Approaches in Current Antiviral Therapy

Viruses cannot reproduce by themselves. They are small (commonly ranging from 20 to 30 nm), obligate intracellular parasites consisting of either double- or single-stranded DNA or RNA as their genetic material, enclosed in an outer shell of a protein called a capsid [1,6]. The virus attaches itself via the glycoproteins on the envelope to the receptor/co-receptor molecules on the host cell membrane, injecting its genetic material into the host cell cytosol. In the next step, the viral genome (DNA or RNA) is transported to the nucleus and incorporated into the genetic material of the host cell, inducing it to replicate the viral genome and messenger RNA (mRNA) molecules. The viral mRNA is translated into structural and regulatory proteins in the cytoplasm, utilizing the host cell protein synthetic machinery. All the necessary components are packed together to produce new viruses. Then, the host cell releases the newly created viruses, either through the lysis of the host cell or by budding off through the cell membrane. The lysis process causes the death of the host cell whereas budding may not [2,28]. Some viruses show remarkable genetic stability and others show a variable rate of mutation [1]. Various antiviral therapy approaches, with their mechanisms, advantages, and limitations, are briefly discussed in this section (and summarized in Table 1) as a prerequisite for the rational development of antivirals and antiviral dosage forms.

### 2.1. Antivirals’ Classification According to the Basis of Their Target

Antiviral therapeutics may be differentiated into three groups: (i) virucides, (ii) immunomodulators, and (iii) antivirals. (i) Virucides are agents capable of neutralizing or destroying a virus; they include phenol, sodium hypochlorite, ethanol, detergents, and ultraviolet rays. (ii) Immunomodulators consist of molecules that augment the host response to infections by promoting the secretion of antibodies or interferons or by intensifying cell-mediated immunity; they include immunoglobulin/antibodies, cytokines, interferons, soluble receptors, receptor antagonists, hormones, cells, cell extracts, low-molecular-weight compounds, antigens, and therapeutic-vaccine-originating DNA. (iii) Antivirals are molecules that inhibit viral multiplication by targeting a particular stage of viral replication such as viral adsorption, fusion, uncoating, reverse transcription, integration, nucleic acid synthesis, and maturation, without causing unacceptable side effects. The antiviral drug should reach the infected organ, inhibit virus function without affecting the host, be readily absorbed, and not be toxic, carcinogenic, allergenic, or mutagenic [1].

Antivirals may be classified (as illustrated in Table 1) into seven groups according to the basis of their target: (i) fusion (attachment) inhibitors inhibit binding/fusion of the virus to the host cell surface and thus prevent cell infection. They target either viral surface proteins (enfuvirtide) or cell receptors (maraviroc) [10]. (ii) DNA/RNA polymerase inhibitors (DPIs/RTIs) block the enzymatic function of viral DNA polymerase/reverse transcriptase, either by binding directly to the active site of an enzyme (nucleoside inhibitors) or binding to allosteric binding sites within the polymerase, thus blocking its action (non-nucleoside inhibitors) and preventing viral replication. DPIs may be nucleoside analogs (idoxuridine, vidarabine, and acyclovir), nucleotide analogs (cidofovir), or pyrophosphate analogs (foscarnet and phosphonoacetic acid) [66]. (iii) Reverse transcriptase inhibitors (RTIs) are nucleoside analogs (zidovudine, didanosine, zalcitabine, stavudine, and lamivudine), nucleotide analogs (tenofovir and adefovir), or non-nucleoside analogs (efavirenz, nevirapine, delavirdine, and etravirine) of endogenous nucleosides and nucleotides. They compete with their corresponding endogenous deoxynucleoside triphosphates for incorporation by HIV reverse transcriptase. Once incorporated, they serve as chain-terminators of viral reverse transcripts, acting on the viral replication cycle by inhibiting a critical step of proviral DNA synthesis before integration into the host cell genome [67]. (iv) Integrase inhibitors are molecules that suppress integrase, an enzyme that facilitates the incorporation of HIV’s proviral DNA into the host cell genome and catalyzes a function vital to viral replication, thus preventing integration of viral DNA into the host genome (raltegravir) [68]. (v) Portmanteau inhibitors are a novel approach where researchers are attempting to design a drug that functions as an RTI as well as an integrase inhibitor [1]. (vi) Protease inhibitors prevent virus replication by inhibiting the activity of viral proteases. They inhibit the cleavage of protein precursors necessary for the production of infectious particles (saquinavir, indinavir, amprenavir, nelfinavir, and ritonavir). (vii) Signaling inhibitors block signaling by interacting with key components of signaling pathways that are involved in viral replication (ribavirin and viramidine are used as inosine monophosphate (IMP) dehydrogenase inhibitors) [1,69].

### 2.2. Limitations on the Use of Antivirals as a Task for Further Development

The benefits and limitations of antivirals classified into particular groups are highlighted in Table 1. Clinical use of the currently available antiviral drugs is limited by their toxic side effects. Toxicity is a major problem with all antivirals as they lack absolute specificity against viruses, resulting in interference with normal cellular functions. For example, many antivirals used against HIV are highly toxic to white blood cells and cause megaloblastic anemia, late drug toxicity with drug-induced immunosuppression, mitochondrial toxicity, and nerve injury [1].

Another limitation is the fact that the more selective the antiviral drug, the narrower its antiviral activity spectrum [4].

The prolonged use of antivirals may result in viral latency. As soon as the antiviral treatment is stopped, the latent virus starts proliferating once more [1]. In addition, since antiviral drugs target virus replication, the latent phases characteristic of some viral (i.e., herpesviral) infections are not amenable to chemotherapy. This is particularly relevant for herpesvirus and retrovirus infections. Thus, eradication of latent virus infections is not feasible currently [4]. There is also the risk of the emergence of drug-resistant virus strains. Variable rates of mutation in viruses result in the generation of viral mutant pools inside the cells, which may lead to alteration in viral enzymes or structural components. The selective pressure caused by antiviral agents under these circumstances results in the replacement of wild-type viruses with mutant ones. The next important issue to be addressed is cross-resistance among antiviral drugs. Resistance to one drug is accompanied by reduced susceptibility to another drug of the same class. To minimize resistance, several measures have been suggested, which include the use of combination therapy and avoiding prolonged/discontinued use of antiviral drugs [1]. 

One of the major challenges associated with antiviral drugs is bioavailability, affected by a drug’s ability to be absorbed by the gastrointestinal tract. For example, in the case of acyclovir, some patients presented absorption rates as low as only 15% of the administered drug. The bioavailability is affected by solubility and permeability. Consequences associated with low bioavailability are a higher required dose, which may lead to toxic effects. The typical delivery method of the antivirals is oral, but researchers have explored topical and intravenous methods to increase bioavailability. These routes present their dangers. Not only is the delivery method important, but many viruses reside in hard-to-access reservoirs such as the lymphatic system or synovial fluid, both of which current antivirals cannot reach [5]. In addition, the half-life of many antivirals is short, which leads to an increased frequency of administration. Frequently administered drugs lead to patient non-compliance and excessive cost [1]. With respect to the above-mentioned facts, the research in antiviral therapy is oriented toward finding new antivirotics or improving those in clinical practice. Another way to enhance antiviral therapy is based on the development of new dosage forms for effective antiviral delivery.

## 3. Dosage Forms and Drug Delivery Systems for Antivirals

A medication administration route is often classified by the location at which the drug is administered, such as oral or intravenous. The choice of routes in which the medication is given depends not only on convenience and compliance but also on the drug’s pharmacokinetics and pharmacodynamic profile [70]. The most common means of drug administration in current antiviral therapy, such as oral, parenteral, and topical (by inhalation, ocular, transdermal, or vaginal), are adopted to achieve a systemic or local medical effect.

Oral administration of medication is most commonly used as it is a convenient, cost-effective medication administration route. However, the bioavailability of medication is influenced by the amount of drug absorbed across the intestinal epithelium. The first-pass effect is an important consideration for orally administered medications, where the drug concentration is significantly diminished before it reaches the systemic circulation, often due to the metabolism in the liver [71]. Antivirotics are slowly and poorly absorbed from the gastrointestinal tract and bioavailability is decreased. Nonetheless, the oral route remains the most preferred thanks to its non-invasiveness, patient compliance, and convenience of drug administration [72].

Intravenous injection is the most common parental route of medication administration and has the benefit of bypassing the first-pass metabolism by the liver. Given their superficial location on the skin, peripheral veins provide easy access to the circulatory system and are often utilized in the parenteral administration of medications [73]. Antivirotics are delivered with a precise dose quickly and in a well-controlled manner throughout the body. Intravenous injection is also used to deliver irritating solutions, which would cause pain and damage tissues if given by subcutaneous or intramuscular injection [28].

An inhaled medication is delivered rapidly across the large surface area of the respiratory tract epithelium. Drugs absorbed into the pulmonary circulation enter directly into the systemic circulation via the pulmonary vein, bypassing the first-pass metabolism. The particle size of the inhaled medication is usually 1–10 µm for effective delivery. The efficacy of drug delivery to the lungs depends not only on the drug particle size and morphology but also on the patient’s respiratory physiology, such as the tidal volume and tracheal inspiration velocity [74]. The inhaled antiviral delivery system offers opportunities for improved dosing, simpler, less invasive administration, enhanced patient adherence, and product life cycle management. The main limitation for patients is necessary respiratory coordination [75]. 

A vaginal route is an underexplored drug delivery route that is not commonly used but has the advantage of bypassing the first-pass effect and can serve as an effective method for local and systemic therapy. The veins from the middle and upper vagina drain directly into the inferior vena cava and bypass the hepatoportal system [70]. Antiviral drugs are easily and rapidly absorbed through the vaginal epithelium into the systemic circulation, and there are no adipose tissue or other cell layers with metabolic enzymes to traverse as with the transdermal or oral routes. The gastrointestinal tract and hepatic first-pass effects are avoided. Disadvantages such as low residence time and discomfort have been surpassed by newly designed drug delivery systems, particularly those based on bioadhesive polymers [76].

**Figure 1:**
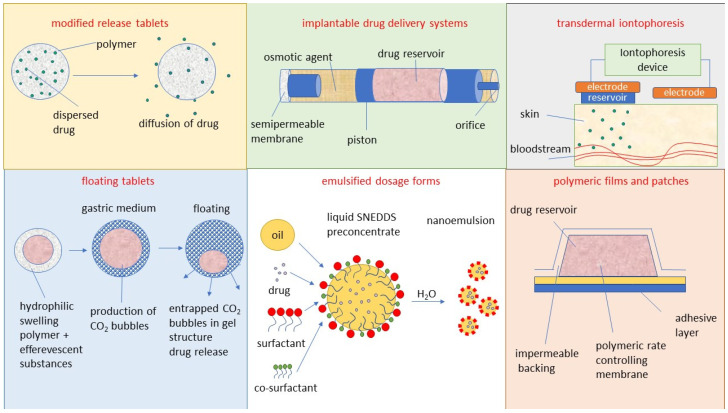
Selected innovative dosage forms for antiviral therapy).

**Figure 2:**
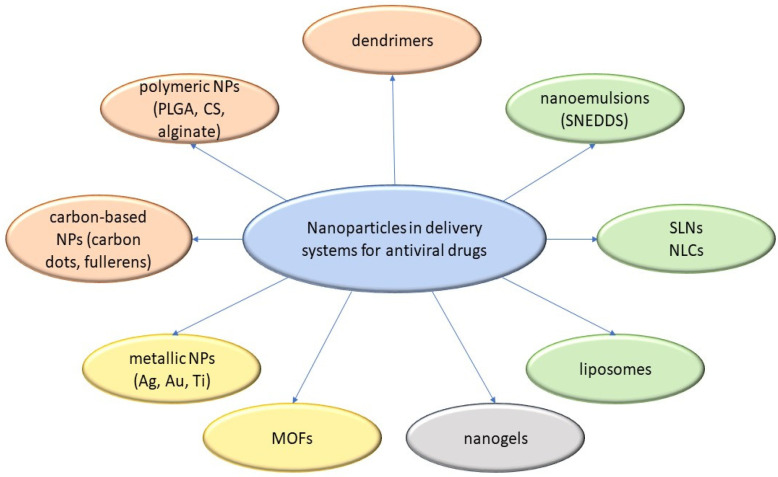
Different types of nanoparticles used as carriers for antiviral drugs.

The transdermal route can deliver drugs through the skin. The common methods of administration through this route are local application formulations such as transdermal ointments and gels, drug carriers such as NPs and liposomes, and transdermal patches [77]. The transdermal delivery of drugs is an attractive approach due to ease of administration, bypassing of the first-pass metabolism, and the large skin surface area. Disadvantages include potential skin sensitization or irritation, discomfort from adhesives, imperfect skin adhesion, cost, and selectivity for specific physicochemical drug properties [78]. The development of new antiviral drugs can be hindered by several factors such as the low efficacy of antiviral agents, the low solubility of the compound, and low bioavailability when administered in the conventional dosage form. Furthermore, some compounds have a short half-life, systemic toxic side-effects, and high cost. An approach oriented toward the development of novel drug delivery can be used to achieve efficacious therapy by improving the design, formulation, and delivery of existing antiviral agents [6]. Innovative dosage forms help to decrease toxicity and dosage and may enhance the stability, specificity, and efficiency of antivirals. The development of innovative dosage forms for antivirals is oriented toward modified-release formulations such as depot tablets, floating systems, implants, or films, as illustrated in Figure 1. In the field of antiviral carriers, the research is devoted to lipid-based NPs including liposomes [10], solid lipid NPs and nanostructured lipid carriers NLCs [12], metallic NPs including gold [79], carbon-based NPs including fullerenes [80], oligomer-based NPs including cyclodextrins [81], polymeric NPs [82] including PLGA, CS, and alginate, dendrimers [83], hybrid NPs including metal-organic frameworks (MOFs) [84], microparticles [85], and micro- and nanoemulsions [86] including self-nanoemulsifying drug delivery systems (SNEDDS) [87], as illustrated in Figure 2. Targeted delivery of antivirals is still of great concern. This helps to decrease toxicity and dosage and may enhance the stability, specificity, and efficiency of antivirals. Carriers such as liposomes and NPs are used for this purpose, but owing to the instability of carriers, rapid release or the bioavailability of antivirals remains a major problem. Certain solutions have been proposed. For example, recently, some novel methods such as the use of heterodimer-loaded erythrocytes with azidothymidine and self-micro-emulsifying systems with acyclovir have shown encouraging results [1]. The following Section 3.1, Section 3.2, Section 3.3, Section 3.4, Section 3.5, Section 3.6 and Section 3.7 discuss the most important conventional as well as innovative dosage forms and delivery systems for antivirals (excluding CS-based systems, which are evaluated separately in Section 4). Mechanisms of action of particular dosage forms and delivery systems are briefly characterized, and illustrative examples are given for particular antivirals along with their potential therapeutic benefits and limitations. Such an evaluation can serve as a basis for the rational development of CS-based antiviral dosage forms and delivery systems (as reported in subsequent Section 4).

### 3.1. Modified-Release Tablets

Since tablets are the conventional dosage form for most drugs including acyclovir (ACV), modifications in such systems are easily acceptable by patients. Possible therapeutic benefits of a modified-release product include improved efficacy and reduced adverse events, increased convenience and patient compliance, optimized performance, a greater selectivity of activity, and new indications. Modified-release formulation design can be conducted for oral and non-oral administration routes [88].

Karpe et al. [89] developed oral disintegration tablets of acyclovir through direct compression and wet granulation methods with the addition of super disintegrants such as sodium croscarmellose and sodium starch glycolate. Tablets containing sodium starch glycolate showed an excellent in vitro dispersion time, with maximum drug release in 10 min.

As a model of a responsive drug delivery system, a magnetic depot tablet was designed by Groening et al. [90] for the oral administration of ACV to prolong gastrointestinal transit. An in vivo bioavailability study was carried out on healthy male volunteers to investigate the influence of extracorporal magnets. To control the release of the active substance, the tablet layers contained hydroxypropylmethylcellulose (HPMC). Two preparations with different amounts of HPMC in the outer coat were developed. A higher amount of HPMC in the outer coat causes a delay in the drug release. By using 10% HPMC, 80% of the ACV dose is released within 5 h. A higher amount of HPMC (15%) in the outer coat leads to a drug release of 80% within 8 h. Additional experiments showed that an external magnet does not influence the release of ACV from depot tablets containing HPMC.

Certain approaches have been also applied to increase the solubility of low-soluble drugs. The complexation of tricyclic ACV derivatives in buffered aqueous solutions of hydroxypropyl-β-cyclodextrin at pH 5.5 or 7.0 has led to a noticeable increase in their solubility at 25 °C and 37 °C. The formation and presence of this inclusion complex were proven by 1H-NMR and differential scanning calorimetry analysis [91]. The complexing capacity of β-cyclodextrin/poly (amidoamine) copolymer with ACV was determined and the antiviral activity of complexed ACV was evaluated against virus cell cultures of HSV type I, for which the complex was found to elicit greater antiviral activity than the pure drug [92].

### 3.2. Floating Delivery Systems

These are low-density systems that float over the gastric contents and remain buoyant in the stomach for a prolonged period without affecting the gastric emptying rate. A gastro-retentive floating drug delivery system is used to delay the residence time of delivery in the stomach. Controlled gastric retention of solid dosage forms may be achieved via mucoadhesion, flotation, sedimentation, expansion, and modified shape systems, or through the administration of pharmacological agents that delay gastric emptying. This involves researchers targeting the release of the drug at a specific site to create systemic or local effects [93].

The single-unit dosage form of floating capsules of ACV was designed by Ahmed et al. [94] using low-density polymers, in which HPMC K4M provided a zero-order sustained release of the drug. The same group tried to develop floating matrix tablets using similar excipients with the addition of Comprito 888 ATO (Gattefosse, Saint-Priest, France) to enable direct compression of the mass, which used a zero-order drug release mechanism [95].

In the work of Vinodbhai et al. [96], multiple-unit floating microspheres were designed using ethyl cellulose and a double emulsion solvent evaporation method, which showed sustained release for 10 h and buoyancy for up to 12 h. Singhal et al. [97] prepared oil-entrapped floating beads using the emulsion-gelation method, in which the percentage of oil played an important role in controlling the floating behavior. The beads containing 20% oil and a 2:1 drug:polymer ratio showed an optimum entrapment efficiency of 89.54% and sustained release for 8 h, with Higuchi model kinetics *n* < 0.5 under fed state conditions.

### 3.3. Implantable Delivery Systems

Mechanisms of drug release from implantable systems are mainly classified into four groups: matrix degradation, controlled swelling, osmotic pumping, and passive diffusion [98]. For systems based on controlled swelling, solvent penetration into the matrix of the device controls the rate of release. This is usually much slower than the diffusion of the drugs, and will, therefore, lead to a lower release rate [99]. Although diffusion from swollen matrices is mainly responsible for drug release, matrix degradation can also contribute to the effectiveness of these systems [100]. Implantable drug delivery devices can be broadly classified into two main groups: passive and active implants. The first group includes two main types of implants: biodegradable and non-biodegradable implants. The second group includes devices such as osmotic pressure gradients and electromechanical drives [101]. Biodegradable implantable drug delivery devices generally consist of a drug reservoir surrounded by a polymer or a drug–polymer mixture. When inserted into the desired area of the body, the drug will be released at a predetermined rate as the polymer degrades. Drug release from a reservoir system is controlled by the rate of polymer degradation or the drug dissolution into and then diffusion through the polymer wall, or a combination of both. Drug release from a drug–polymer mixture is controlled by diffusion, swelling, or erosion. The release of the drug from the system will be dependent on the solubility and permeability of the drug in the polymer, the drug load, and the in vivo degradation rate of the polymer [102]. The degradation time of polymers can vary extensively depending on features such as the polymer molecular weight and surface properties [103]. This will affect the release of any drug contained within a formulation. In addition, degradation will also be dependent on in vivo factors such as pH and temperature [102].

Matrix-type ocuserts of ACV were fabricated by Alam et al. [104] using water-soluble polymers such as polyvinyl alcohol and methyl cellulose via the film casting method, in which the rate and drug release profile were easily modified by varying the additives. In another matrix-type implant, the ACV–cyclodextrin complex was dispersed in an HPMC medium and then sandwiched between cellulose acetate phthalate to control the rate of drug release. This product remained stable with a shelf life of 1.8 years. The in vitro release was found to be favorable with 5% cellulose acetate phthalate in the membrane, and the in vivo evaluation carried out in rabbits showed a significant in vitro/in vivo correlation with the release studies [105]. 

Reservoir-type ocular inserts were developed by Shanmugam et al. [9] with HPMC K4M, polyvinylalcohol, sodium alginate, Eudragit RL 100, and Eudragit RS 100, which showed zero-order release and were found to be stable, sterile, and nonirritating. Another implant containing 2.5% sodium alginate with a 3.5:1.5 ratio of Eudragit RL 100 and RS 100 in vivo showed the presence of 1.7 μg/mL ACV in the aqueous humor after 8 h, which remained for up to 5 days and which supports the notion of a high in vitro/in vivo correlation [106]. In the study by Deshpande et al. [107], ACV was incorporated as a binary system with β-cyclodextrin and dispersed into HPMC, and was made the reservoir for the implant. The release was controlled for up to 20 h with non-Fickian diffusion behavior and high in vitro/in vivo correlation in the release rates.

### 3.4. Transdermal Iontophoresis Delivery Systems

Transdermal drug delivery is a painless method of delivering drugs systemically by applying a drug formulation on intact and healthy skin [108,109]. The drug initially penetrates through the stratum corneum and then passes through the deeper epidermis and dermis without drug accumulation in the dermal layer. When a drug reaches the dermal layer, it becomes available for systemic absorption via dermal microcirculation [110,111]. Transdermal drug delivery has many advantages over other conventional routes of drug delivery [110,112,113]. It can provide a non-invasive alternative to parenteral routes, thus circumventing issues such as needle phobia [108]. A large surface area of skin and ease of access allow for many placement options on the skin for transdermal absorption [109]. Furthermore, the pharmacokinetic profiles of drugs are more uniform with fewer peaks, thus minimizing the risk of toxic side effects [108]. In addition, transdermal drug delivery can improve patient compliance due to the reduction in dosing frequencies and is also suitable for patients who are unconscious or vomiting, or those who rely on self-administration [114]. Transdermal drug delivery avoids pre-systemic metabolism, thus improving bioavailability [108,115].

The potentialities of the application of iontophoresis were investigated for facilitated transdermal delivery and passive diffusion across nude mouse skin for ACV individually and in addition to penetration enhancers such as cetrimide and sodium lauryl sulfate, where the former showed threefold greater permeation than the later [116]. In the same fashion, the in vivo efficacy of controlled transdermal delivery of ACV (topical and systemic antiviral efficacy) was evaluated using hairless mice models for cutaneous HSV-1 infections. This study demonstrated as a distinctive example of dose/flux-response correlation in local antiviral therapy since the typical sigmoidal curve of reasonably high reproducibility was obtained for the antiviral efficacy versus the rate of drug release [117].

### 3.5. Polymeric Films and Patches

Films are thin membranes containing the drug in a matrix or reservoir, which can be utilized to amend the extended release of the drug at varying sites such as topical, ocular, dental, buccal, nasal, vaginal, rectal, etc. These films can be made erodable or nonerodable depending on the type of polymers employed for the design with respect to the site and drug release required [7]. By utilizing the inclusion phenomenon, a buccal patch was designed by Saxena et al. [118] with hydrophilic polymer hydroxypropyl beta-cyclodextrin, which showed a substantial increase from 64.35% to 88.15% ACV release, confirming the success of inclusion complexes for the formulation of a buccal patch of ACV.

In another study, by employing the solvent evaporation method, thin films of poly (ethylene-co-vinyl acetate) copolymer matrix were designed for ACV and chlorhexidine combination therapy. The outcome of the drug combination using varying copolymer compositions and the effect of coating were studied for their suitability in the oral environment. The increase in the concentration of vinyl acetate increased the drug release rate whereas the coating of films decreased the release rate [119].

### 3.6. Emulsified Dosage Forms

Emulsified systems containing a dispersion of oil and water with the addition of surfactants have shown specific advantages in the delivery of poorly soluble drugs and in masking the bitter taste of the active pharmaceutical ingredients (APIs). Different types of microemulsion, nanoemulsion, and self-emulsified dosage forms (SNEDS) were designed for the controlled delivery of ACV [7]. For the oral route of administration, a microemulsion developed with labrasol and plurol oleique as surfactant and cosurfactant, respectively, showed an increase in bioavailability when compared with the commercially available tablets [120]. In the same way, a novel microemulsion-based topical formulation of ACV resulted in total inhibition of herpetic skin lesions [121]. A liquid-in-oil microemulsion system containing a 3:2 ratio of Tween 80 and Span 20 as nonionic surfactants and dimethyl imidazolium dimethyl phosphate as the pseudo phase was identified to show excellent solubility and a skin permeation enhancing effect for ACV when used in a model of a sparingly soluble drug with Yucatan micropig porcine skin. Low cytotoxicity was reported for the carriers when using the reconstructed human epidermal model LabCyteTM EPI-MODEL12 [122].

### 3.7. Micro- and Nanoparticulate Delivery Systems

Unlike conventional dosage forms, micro- and nanoparticulate drug delivery systems, thanks to their small size and efficient carrier characteristics, offer numerous advantages compared to conventional dosage forms, which include improved efficacy, reduced toxicity, improved patient compliance, and convenience. These drug delivery systems can sustain drug action at a predetermined rate, are relatively constant (zero order kinetics), produce an efficient drug level in the body, and simultaneously minimize undesirable side effects. They can also localize drug action in the diseased tissue or organ through targeted drug delivery using carriers or chemical derivatization. Particulate-type carriers, also known as colloidal carrier systems, includes lipid particles, micro- and NPs, micro- and nanospheres, polymeric micelles, and vesicular systems such as liposomes, sphingosomes, niosomes, transfersomes, aquasomes, ufasomes, and so forth [123]. Chen et al. [124], in their review article, discussed several types of NPs, such as quantum dots, gold, silver NPs, nanoclusters, carbon dots, graphene oxide, silicon NPs, polymers, and dendrimers, possessing remarkable antiviral ability. Despite their difference in antiviral mechanism and inhibition efficacy, these functional nanoparticle-based structures have unique features as potential antiviral candidates. Figure 2 shows the different types of NPs used as carriers for antiviral drugs.

*Liposomes*. Various bioavailability studies of niosomes and liposomes have been reported with remarkable improvement in drug delivery of antiviral drugs. In vivo oral bioavailability of niosomal delivery of ACV was investigated using rabbit models and was improved when compared with the free solution with evidence of an increase in the mean residence time [125]. The bioavailability of ACV formulated in liposomal mucoadhesive gel administered through the intranasal route was 60.72%, which was comparable with the intravenous route [126]. Ocular pharmacokinetics of ACV encapsulated in liposomes compared to an ointment showed that the aqueous humor concentration and area under the curve were significantly higher in the liposomes [127]. Garg et al. [128] prepared atazanavir-encapsulated galactosylated liposomes to target the lectin receptors present on macrophages. The drug delivery system resulted in increased uptake by alveolar macrophages and better drug distribution in the lymph nodes, liver, spleen, and lungs compared to free atazanavir. In addition, no toxicity was observed after intravenous injection in rats, confirming that the galactosylated liposomes might offer a potential system for targeted drug delivery.

*Solid-lipid NPs (SLNs)* and *nanostructured lipid carriers (NLCs)* have been used for the encapsulation and delivery of various types of antiviral agents. Ritonavir has been encapsulated in SLNs and shown to exhibit sustained release properties and antiviral activity in an in vitro anti-HIV-1 model [129]. In the work of Seyfoddin et al. [130], both SLNs and NLCs were investigated for their ability to increase the ocular bioavailability of ACV. The NLCs were shown to have a higher encapsulation efficiency and efficacy than the SLNs, which was attributed to the less regular lipid crystalline structure.

**Figure 3:**
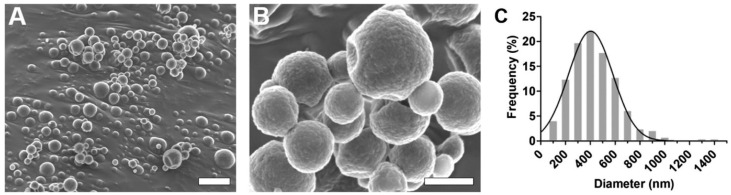
Representative SEM images of TDF/FTC-NPs at the magnifications of (**A**) 15,250× (bar = 2 µm) and (**B**) 100,000× (bar = 0.5 µm). (**C**) Particle diameter distribution determined by SEM and corresponding Gauss fitting [82]. Reproduced with permission from Elsevier (Copyright © 2019).

*Polymeric nanoparticles*. Polymeric nanoparticles are preferably made up of polymers obtained from natural, synthetic, or semi-synthetic sources, which may be either biodegradable or non-biodegradable. Among synthetic polymers, Eudragit, an acrylate derivate, is used for the production of controlled-release (sustained and gastroresistent) nanoparticulate systems. [14]. In the work of Cautela et al. [82], different types of films containing the antiretroviral drugs tenofovir disoproxil fumarate (TDF) and emtricitabine (FTC) were developed. Formulations based on poly(vinyl alcohol) and pectin were produced as single- or double-layered films containing TDF/FTC or TDF/FTC-loaded Eudragit® L 100 NPs. Representative SEM images of TDF/FTC-NPs are shown in Figure 3. Double-layered films significantly reduced the burst effect and the overall release of both drugs compared to fast-release, single-layered films. The effect of delaying drug release was most noticeable when TDF/FTC-loaded NPs were incorporated into double-layered films. This last design seems particularly advantageous for the development of a coitus-independent, on-demand microbicide product. Moreover, all film types were shown to be potentially safe when evaluated via MTT metabolic activity and lactate dehydrogenase release assays using HeLa and CaSki cervical cell lines.

*Cyclodextrin-based NPs.* Cyclodextrin (CD) inclusion complexes have been mainly and successfully used in the pharmaceutical field for enhancing the solubility, dissolution rate, and bioavailability of several poorly soluble drugs. A combination of drug complexation with CDs and complex incorporation into NPs is a possible strategy to overcome the drawbacks associated with each separate system, and then improve their effectiveness, by combining their respective beneficial effects in one carrier [131]. Recently, five new NPs were synthesized and characterized by Perret et al. [132]. They investigated their capacity to form NP in water and encapsulate ACV with high loading and sustained release. In another work concerning ACV, Lembo et al. [81] designed cyclodextrin-based nanosponges (Carb-NS) carrying carboxylic groups within their structure as a new ACV carrier. The ACV loading into Carb-NS was higher than that obtained using NS, reaching about 70% (*w*/*w*). In vitro release studies showed the release kinetics of ACV from Carb-NS to be prolonged in comparison with those observed with NS, with no initial burst effect. The NS uptake into cells was evaluated using fluorescent Carb-NS and revealed NP internalization. Enhanced antiviral activity against a clinical isolate of HSV-1 was determined using ACV loaded in Carb-NS.

*Dendrimers.* Dendrimers are highly branched structures with repetitive sequences of monomers called dendrons. Dendrimers have three main components: (a) a core moiety, (b) branching units, and (c) surface groups [133]. Dendrimers can have a polymeric nature, e.g., PAMAM (polyamidoamine) [134], or may be based on repetitive units of amino acids [135], even conjugated with lipids [136]. The diameter of a dendrimer is nanosized, similar to certain globular proteins. Dendrimers are considered to be biomimetics of synthesized proteins, but they have significantly better stability (protease resistance); greater lack of complex beta-sheets, coils, and loops of proteins; and a better intrinsic ability to bind drugs through their well-defined internal cavities and surface functions [137]. Dendrimers have been shown to have unique intrinsic antimicrobial properties and also antiviral activities (PAMAM, polylysine dendrimers) [138], for example, against the influenza virus (carbosilane dendrimer uniformly functionalized with carbohydrate moieties) [139], human immunodeficiency virus (polysulfonate dendrimers) [140], and respiratory syncytial virus (peptide polyanions) [141]. Dendrimers have different functional groups on their surfaces and can block the entry of a virus into cells either through cellular protection or their direct effects on virus particles [142].

Kandeel et al. [83] studied the structure–activity relationship of cationic and anionic dendrimers against MERS-CoV using 16 dendrimers with various generations and terminal charges. Three types of polyanionic dendrimers comprising the terminal groups sodium carboxylate, hydroxyl, and succinamic acid and polycationic dendrimers containing primary amine were assessed for their antiviral activity with a MERS-CoV plaque inhibition assay. The dendrimers at a final concentration of 10 µM were mixed with MERS-CoV before the infection of Vero cells. Most dendrimers had a variable degree of inhibitory activity on plaque formation. Results showed that polyanionic dendrimers can be added to antiviral preparations to improve the delivery of antivirals, as well as the intrinsic antiviral activity.

Dendrimers can be used both as direct inhibitors of HIV and as drug delivery vehicles. For example, Han et al. [143] designed a polylysine dendrimer containing sulfated cellobiose end groups. It was found that the sulfated cellobiose dendrimer 4 had potent anti-HIV activity with an EC_50_ value (half maximal concentration value) comparable to that of dideoxycytidine (anti-AIDS drug), a reverse transcriptase inhibitor. It also showed intermediate anticoagulant behavior similar to that of curdlan and dextran sulfate. These biological activities might originate from cluster effects based on the dendritic structure.

In other studies focused on HIV-1 therapy, dendrimers have been designed to inhibit membrane fusion (e.g., dendrimers with multivalent carbohydrate terminal groups) [144] or disrupt HIV-1 capsid assembly (e.g., gallic acid–triethylene dendrimers) [145].

*Nonpolymeric nanoassemble*. A nonpolymeric nanoassembly type for ocular drug delivery of ACV was designed by Stella et al. [146] and its in vivo pharmacokinetic performance in rabbits was evaluated. For this purpose, a new lipophilic derivative of ACV was synthesized, possessing greater lipophilicity and supporting the formation of a homogeneous water dispersion with a higher amount of ACV than the aqueous solution of the parent drug. This was achieved by chemically linking ACV to the isoprenoid chain of squalene, thus obtaining 4’-trisnorsqualenoylacyclovir, in which squalene is covalently coupled to the 4’-hydroxy group of acyclovir. This new prodrug was then formulated as nonpolymeric nanoassemblies through nanoprecipitation. The pharmacokinetic profile of the nanoassemblies in tear fluid and aqueous humor showed increased absorption of the drug compared to a free drug solution.

*Metallic NPs.* Of the inorganic NPs, silver NPs (AgNPs) have shown promising antibacterial and antiviral efficacy. AgNPs can bind viral surface proteins and inhibit the interaction between viruses and cell membrane receptors. It has also been reported that AgNPs can deactivate viruses by denaturing surface proteins containing cysteine and methionine residues on the viral capsid [21]. Lin et al. [147] designed zanamivir-modified AgNPs (Ag@ZNV) for the inhibition of H1N1 virus neuraminidase activity. Moreover, Ag@ZNV inhibited caspase-3-mediated apoptosis through reactive oxygen species (ROS) production, confirming that Ag@ZNV could effectively reduce apoptosis due to H1N1 infection.

However, there is increasing concern about the possible impact of AgNPs on the environment and, subsequently, human health. It is still not clear to what extent the intact AgNPs themselves can enter the human body, whether the AgNPs change the physiological environment if the Ag+ ions released from the NPs are absorbed, or if the effect observed is due to the AgNP-induced inflammatory response, the ions released, or the nanoparticulate form itself [148].

Gold NPs (AuNPs) are less toxic to healthy cells and more conducive to translation to clinical applications than AgNPs. In addition, AuNPs could inhibit virus entry into host cells by interacting with hemagglutinin and oxidizing the disulfide bond of this glycoprotein, leading to its deactivation and thereby hindering the membrane fusion of the host cell with the virus [21]. Kim et al. [79] showed that porous AuNPs were more effective in inhibiting influenza A virus infection than nonporous AuNPs. This was related to the higher surface area of the porous material, with the higher surface area facilitating the interaction of AuNPs with the envelope, thereby increasing their antiviral activity.

*Fullerenes*. Fullerenes, which are the earliest discovered carbon-based nanostructures, have been researched as potential antiviral treatments. One application that is commonly associated with this specific material is antiviral treatment against HIV by blocking encoded enzymes through the inhibition of active sites on the HIV protease. Other promising applications of fullerenes have been reported in antiviral therapy of diseases caused by hepatitis C virus (HCV), respiratory syncytial virus (RSV), H1N1, herpes simplex virus, human cytomegalovirus, Zika, and Dengue viruses [5]. Muñoz et al. [80] synthesized spherical topological molecules formed from hexadecane adducts of fullerenes by introducing 120 sugar units using efficient CuAAC click chemistry, with the centrally located fullerene molecule covalently linked to 12 surrounding fullerene molecules, each of which was sequentially assigned 10 monosaccharides. Results showed that these nanospheres effectively inhibited artificial Ebola virus infection of cells and suppressed virus concentrations to half of the subnanomolar range.

*Hybrid NPs.* Hybrid NPs are used to create delivery systems for antivirals by combining the beneficial attributes of different individual NPs. For example, hybrid NPs can be formulated by trapping smaller particles inside larger particles, or by forming core–shell structures, which can lead to improved encapsulation, protection, or release properties [19]. Ramanathan et al. [149] investigated the capability of hybrid NPs composed of a hydrogel core and a lipid shell for improving the antiviral activity of tenofovir disoproxil fumarate (TDF) and maraviroc (MVC) toward HIV. Both MVC and TDF were incorporated within these lipogel NPs, resulting in sustained release of drugs. The hybrid NPs were shown to be effective for antiviral delivery via the vaginal route using an in vivo female mouse model.

*Metal–organic frameworks (MOFs).* MOFs are microporous materials that seem promising for many applications because of their easy synthesis and large variability [150]. MOFs consist of metal clusters, also called secondary building units, and bridging organic ligands, also called linkers [151,152]. Because of the broad variety of metals and linkers, MOFs are materials with highly tunable properties [153,154]. MOFs can be regarded as optimal drug delivery materials due to the possibility of adjusting the framework’s functional groups and tuning the pore size. Even though the major interest in MOFs has been in the area of high-density gas storage for potential use in separations, fuel cells, and other energy-related applications, recent reports suggest that MOFs may have a significant role in drug delivery [155].

Antiviral drug delivery using MOFs has been studied by Agostoni et al. [156], who investigated the use of a MOF obtained by reacting Fe(III) with trimesic acid as an anti-HIV azidothymidine triphosphate (AZT-TP) drug delivery system. The prepared nanoMOFs were able to act as efficient molecular sponges, quickly absorbing up to 24 wt% of AZT-TP with entrapment efficiencies close to 100%, without perturbation of the supramolecular crystalline organization. The results showed that contrary to free AZT-TP, the loaded nanoMOFs efficiently penetrated and released the drug inside major HIV target cells, efficiently protecting against HIV infection.

Horcajada et al. [84] used nano-scaled iron(III) carboxylate, which was converted to MOFs. They then inserted the antiviral and antitumor drugs (busulfan, azidothymidine triphosphate, cidofovir, and doxorubicin) into the iron(III) carboxylate framework. The effect of these nano-vessels was observed on mice. Results showed that the prepared MOFs functioned as superior nanocarriers for efficient controlled delivery of challenging antitumoral and retroviral drugs against cancer and AIDS. In addition to their high loading, they also potentially associate therapeutics with diagnostics, thus paving the way for theranostics or personalized patient treatments.

Akbari et al. [157] examined a novel framework containing TiO_2_@Chitosan@ZIF-8 for the delivery of ACV. The obtained results demonstrated that 90% of the ACV was loaded through the TiO_2_@Chitosan@ZIF-8 framework due to the porous structure, high surface area, and cavities in the structure of the nanocarrier. The drug release study was performed in PBS (phosphate buffer saline, pH:7.4) and AB (acetate buffer, pH:5) solutions, which showed 78% and 82% release within 3 days, suggesting the controlled release of ACV from MOFs attributed to CS.

*Nanoemulsions.* Nanoemulsions are a type of colloidal system that has great potential for the encapsulation, protection, and delivery of drugs. Nanoemulsions can be distinguished from conventional emulsions by their relatively small droplet dimensions (<200 nm). Unlike microemulsions, they are thermodynamically unstable colloidal dispersions. They are typically formed from oil, water, surfactants, and water-soluble co-solvents. Nanoemulsions are classified according to the structural organization of phases into the oil-in-water (O/W) and water-in-oil (W/O) types (more sophisticated types such as oil-in-water-in-oil (O/W/O) or water-in-oil-in-water (W/O/W) nanoemulsions can also be prepared) [19]. A single nanoemulsion system can solubilize and deliver both hydrophilic and lipophilic drugs. It can protect the drugs from hydrolysis and oxidation and improve the bioavailability of the formulation. As a result of its thermodynamic and kinetic stability, nontoxicity, and nonirritant nature, nanoemulsions can be formulated as foams, creams, liquids, and sprays and administered through oral, nasal, pulmonary, enteric, topical, transdermal, and intravenous routes [158]. Mohammadi et al. [159] developed and evaluated nanoemulsions containing ACV for ophthalmic drug delivery. Based on solubility studies, Tween 20, Triacetin, and Tramsectol^®^P were chosen to prepare formulations. Results showed that the prepared nanoemulsions possessed desirable physiochemical properties, including a droplet size of less than 15 nm. Selected formulations (F1 and F2) exhibited a sustained drug release pattern compared to the control group (*p* < 0.001). ACV penetration from F1 and F2 to the excised bovine cornea was 2.85- and 2.9-fold better than the control, respectively. Furthermore, HET-CAM and the modified Draize test confirmed that F1 and F2 were safe for ocular administration.

In the work of Nemade et al. [160], the researchers tried to improve the bioavailability of the antiretroviral drug tenofovir disoproxil fumarate and minimize the side effects of this therapy, which are observed to be on the higher side in chronic HIV treatment. They developed a stable nanoemulsion with a ternary phase system prepared with a combination of surfactants and co-surfactants, to be delivered via the nasal route directly to the brain. Results from ex vivo studies revealed that the developed nanoemulsion (B2) possessed a higher rate of drug release compared to other formulations.

*Self-nanoemulsifying drug delivery systems (SNEDDS).* SNEDDS are widely used to enhance the solubility and bioavailability of drugs with poor aqueous solubility. On dilution with a physiological medium, SNEDDS produce nano-sized drug-loaded oil droplets that are primarily responsible for improved absorption and bioavailability [161], which are less affected by the presence or absence of food [162]. Furthermore, SNEDDS offer high physical and chemical stabilities and can be converted into other dosage forms, such as tablets and capsules [163]. Hosny et al. [87] combined the benefits of penciclovir (PV) and lavender oil (LO), which exhibits anesthetic activity, in the form of SNEDDS for the treatment of herpes labialis. The optimized PV-LO-SNEDDS was embedded in CS hydrogel and the resulting formulations coded by O3 were prepared and evaluated. The rheological studies demonstrated a combined pseudoplastic and thixotropic behavior with the highest flux of PV permeation across sheep buccal mucosa. Compared to a marketed 1% PV cream, the O3 formulation exhibited a significantly higher and sustained PV release, nearly twice the PV permeability, and a relative bioavailability of 180%. Overall, results confirmed that the O3 formulation can provide an efficient delivery system for PV to reach oral mucosa and subsequent prolonged PV release. Thus, the PV-LO-SNEDDS embedded oral gel is promising and should be further evaluated in clinical settings to establish its therapeutic use in herpes labialis.

Khan et al. [164] developed and optimized a SNEDDS for the antiretroviral protease inhibitor drug lopinavir (LPV) (used to treat HIV-1 infection) to increase its limited oral bioavailability (attributed to its poor aqueous solubility, low efficacy, and high first-pass metabolism). The titration method was used to prepare LPV-loaded SNEDDS (LPV-SNEDDS). The resulting LPV-loaded SNEDDS released nearly 99% of the LPV within 30 min, which was significantly (*p* < 0.05) higher than the LPV suspension in methylcellulose (0.5% *w*/*v*). This finding indicates the potential use of SNEDDS to enhance the solubility of LPV, which eventually could help improve the oral bioavailability of LPV. The Caco-2 cellular uptake study showed a significantly (*p* < 0.05) higher LPV uptake from the LPV-SNEEDS than the free LPV (LPV suspension).

The findings discussed in Section 3.1, Section 3.2, Section 3.3, Section 3.4, Section 3.5, Section 3.6 and Section 3.7 demonstrated the efficacy of currently studied formulations of the 2nd and 3rd generations in antiviral therapy.

In this context, it can be summarized that modified-release dosage forms (2nd generation) such as tablets, films, transdermal patches, floating systems, or implants containing antiviral drugs show pronounced advantages when compared with conventional (immediate-release or 1st generation) dosage forms. They mainly contribute to controlling the drug release, i.e., prolonged, sustained, or delayed. In this manner, the bioavailability of the drug is increased, and adverse effects, dosing frequency, and toxicity are reduced. Antiviral drug delivery systems (3rd generation), including nanoparticles and nanoemulsions, are also able to control the drug release and reduce the dose, and, in addition, they improve the solubility of low-soluble drugs, thus improving bioavailability, and they are able even to target damaged tissues, organs, or viral parts. 

## 4. CS-Based Dosage Forms and Drug Delivery Systems for Antivirals

This section is focused specifically on CS-based dosage forms and drug delivery systems for antivirals. Therefore, the properties and preparation of CS and its derivatives, used either as antivirotic agents themselves or to formulate antivirals, are briefly discussed in Section 4.1. Subsequently, CS and its derivatives in nanoparticulate antivirotic drug delivery systems are discussed and evaluated in Section 4.2. The properties and preparation of CS and its derivatives as well as their nanoparticulate systems were comprehensively reviewed for various drugs and drug delivery systems by Mikušová and Mikuš [16]; therefore, general characterization and preparation procedures are considerably reduced here and focused just on antivirals. Instead of a general description, a more detailed evaluation of individual innovative antiviral systems concerning their mechanisms of action, benefits, limitations, and practical implementations is given here.

### 4.1. CS and Its Derivatives as Polymeric Systems in Antiviral Use

#### 4.1.1. Properties, Activities, and Interactions of CS

**Figure 4:**
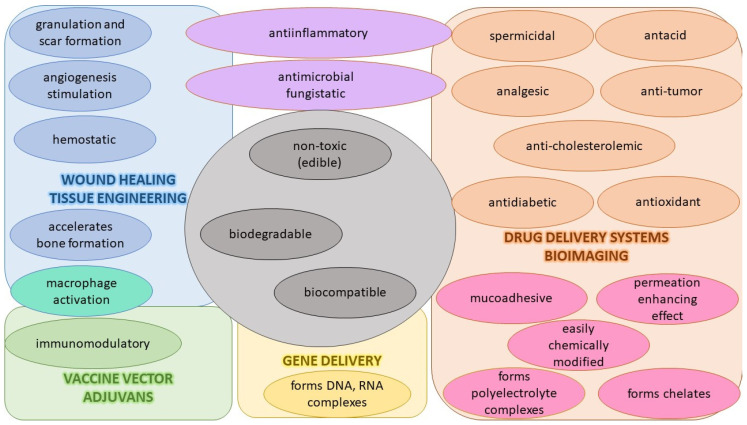
Properties of chitosan and its biomedicinal applications.

Among the polymers used for the preparation of NPs designed for drug delivery of antivirals, CS is frequently chosen. CS has suitably positioned functional groups that confer specific properties to this polysaccharide. Recent review papers focused on CS properties [165,166,167], the relationship between its chemical structure and biological properties [167], preparations [167], general applications of CS [165,166], pharmaceutical applications [168,169] especially in drug delivery [167,168], and the mechanism of drug release [166]. The advantageous properties of CS with potential applications in pharmacy and medicines are illustrated in Figure 4.

Chitin and its deacetylated derivative, CS, are a family of linear polysaccharides composed of varying amounts of (β1→4) linked residues of *N*-acetyl-2 amino-2-deoxy-D-glucose (glucosamine, GlcN) and 2-amino-2-deoxy-D-glucose (*N*-acetyl-glucosamine, GlcNAc) residues [165]. The presence of the amino group at the C-2 position of the glucosamine unit strengthens the functional and structural properties of CS. This amino group represents its cationic nature and imparts inherent properties of wound healing, antimicrobial activity, and most importantly, mucoadhesiveness, making it a good carrier material in drug delivery systems. It has a pKa of 6.5, and it is insoluble in water but soluble in aqueous acidic media. It is protonated and polycationic in nature and forms complexes with diverse anions such as lipids, proteins, DNA, alginate, pectin, and poly(acrylic acid). The physicomechanical properties (solubility, toxicity, hydrophobicity) of CS depend on the degree of deacetylation and molecular weight of CS, which depend on the source of chitin [23].

The mucoadhesive property of CS is attributed to its cationic character. The mucous membrane is composed of mucin glycoprotein and has anionic functionalities in the form of sialic acid and sulfonic acid. The cationic group in CS and these anionic acids in the mucous result in ionic interactions conferring mucoadhesive attributes to CS. The mucoadhesion increases with the degree of deacetylation and its molecular weight and decreases with an increase in crosslinking. This attribute promotes its adherence to the surface of the gastrointestinal tract and upper respiratory tract and is a tool in retaining this carrier for a prolonged time, achieving sustained release of drug payloads [170].

CS, being positively charged and engaged in interaction with the mucus membrane, opens the tight junctions between the cells (by reducing the transepithelial electrical resistance), promoting passage through the mucosal cells and enhancing drug permeation. This is beneficial for hydrophilic and high-molecular-weight compounds such as proteins and peptides. Modified CS such as thiolated and trimethyl CS shows an improved permeation enhancement effect compared to original CS [24].

CS exhibits antibacterial activity due to the positively charged amine groups that interact with the negatively charged components of the bacterial cell walls. The antimicrobial properties are primarily confined to a pH below 6. This behavior can limit applications and investigations under neutral and alkaline conditions; chemical modification of functional groups can give CS derivatives enhanced bioactivity and improved aqueous solubility, which maintains the inherent properties of CS as well as magnifies the scope of application. Other mechanisms of antimicrobial effect are: (i) a formation of a film on the porins of the cell surface to block the exchange of nutrients, leading to microbial cell death; (ii) CS penetration of the cell wall to affect DNA/RNA and protein synthesis; (iii) chelation of metal ions by unprotonated amino groups of CS on the cell surface to disrupt cell walls or membranes [171]. CS is active against both Gram-positive and Gram-negative bacteria and is used in biomedical and clinical science to avoid bacterial colonization. It possesses antifungal activity wherein the mechanism of action involves morphogenesis of the cell wall, directly interfering with fungal growth. Low-molecular-weight CS can pass through the cell membrane and interact with DNA to interrupt its functions. The antibacterial activity of CS depends on the pH of the environment, type of pathogen, degree of deacetylation, molecular weight, and concentration [172,173].

CS exhibits very good biocompatibility because of its structural and functional resemblance to glycosaminoglycans present in the extracellular matrix of the human body and quickly forms hydrogels through crosslinking methods. It is easily degraded by in vivo lysozymes, chitinases, and colon-residing bacteria through the cleavage of glycosidic linkage in its structure. These properties can prove extremely beneficial for the development of biocompatible and biodegradable drug delivery systems [174].

The ability of CS to form ionic crosslinks leads to the formation of stable complexes, releasing the drug over a prolonged period and conferring controlled drug release. This is beneficial for drugs that show suboptimal plasma levels through oral administration and have to be given parentally. It is also useful for carrying drugs that are susceptible to metabolic degradation in the gastrointestinal tract, ensuring enhanced efficacy and patient compliance [175].

Cleavage of glycosidic bonds leads to the production of oligomers with important variations in the degree of polymerization (chain length). These low-molecular-weight oligosaccharides, known as chitooligosaccharides, abbreviated to COS or oligoCS (CS oligomers, chitooligomers), present low viscosity and relatively small molecular sizes, which, in turn, make them water soluble and confer them versatile biological activities such as ready adsorption in vivo and cholesterol-lowering, antibacterial, and antitumor effects [176]. They are also soluble in solutions at acid and alkaline pHs. Indeed, these products have a remarkably wide range of biological activities and important potential for numerous industrial applications. In target applications such as biomedicine, cosmetics, and agriculture, e.g., as an antibacterial agent or plant growth stimulator, low-molecular-weight CS and oligomers are more effective than high-molecular-weight CS [177,178].

The use of CS polymers in drug delivery also has a weak point. The variability of the CS source and process of preparation lead to the production of a wide range of CS polymers with different physicochemical properties, such as different degrees of deacetylation, molecular weight, crystallinity, and residual ash and protein contents. Such variability can lead to inconsistent and conflicting reports regarding their antiviral performance as well as discrepancies between observations in physicochemical properties and biological actions of polymeric as well as nanoparticulate systems prepared from CS and its derivatives and used in antiviral therapy. In this respect, well-defined oligo CS systems could have benefits over their polymeric counterparts to some extent.

Furthermore, a few studies on the antiviral activity of CS in animals have been reported, some of which have demonstrated that CS itself has antiviral activity against human cytomegalovirus strain AD169, influenza A virus, Rift Valley Fever virus (RVFV), herpes simplex 1 (HSV-1), and Coxsackie viruses [179]. However, there are some other studies indicating a negligible antiviral activity of native CS but significant antiviral activities of several CS derivatives, as is presented in the following subsections of Section 4.1.

#### 4.1.2. Preparations, Modified Properties, and Use of CS Derivatives

Unlike chitin, CS exhibits a limitation in its solubility in water and reactivity. The poor solubility of unmodified CS in organic solvents also limits its utilization. Therefore, the frequent aim of new CS derivatives is to improve their water solubility. At present, there are generally two considered methods for improving the solubility of CS: (i) chemical modification, where a hydrophilic group is introduced on an amino group or a hydroxyl group in a CS molecule. At the same time, this method destroys the original hydrogen bond and crystallinity of CS; (ii) CS degrades into a water-soluble product of small molecules under the action of an enzyme (however, the molecular weight distribution of CS during the degradation is extremely uneven and the product is difficult to separate) [26].

Hence, CS derivatives are prepared to achieve required/favorable chemical, physical, and biological properties, as is discussed in subsections concerning the antiviral action or antiviral formulations. CS derivatives are classified primarily according to the type of covalently bound functional groups. In addition to these structures, various chemically crosslinked (derivatized) CS polymer associates represent (are considered as) new CS structures with modified properties, too. CS has three types of reactive functional groups: two hydroxyl groups on C-3 and C-6 in each repeating unit and one amino group on C-2 in each deacetylated unit. These groups allow the conjugation of many substituents, resulting in new modified derivatives. The main reactions are quaternization (useful to increase the solubility of CS in neutral water), acetylation, reductive amination, acylation, phosphorylation, Schiff’s bases, and crosslinking modifications [180]. The main derivatives are quaternary ammonium CS salts, carboxymethyl-CS, carboxyalkyl-CS, arylCS, hydroxyalkyl-CS, sulfated derivatives, phosphorylated CS, succinyl-CS, and thiolated CS [181,182]. A very recent review by Tan et al. [183] deals with specific molecules of CS derivatives possessing antiviral properties. CS derivatives that were applied just as antivirotics or for the formulation of antivirals are summarized in Table 2 with brief comments on their properties, preparation (derivatization) conditions, benefits, limitations, and uses (see Section 4.1.2).

##### Quaternized CS

The advantages of quaternized CS (QCS) derivatives are their effectiveness as an absorption enhancer in the specific pH close to its pKa and that they have better hydroxyl radical scavenging activity in comparison to other CS. The mucoadhesive property decreases with an increased degree of quaternization. The quaternization of CS involves the insertion of a hydrophilic group via any of three methods: direct quaternary ammonium substitution, epoxy derivative open loop, and N-alkylation. A high degree of substitution provides better aqueous solubility, permanent positive charge, and enhanced antimicrobial action, and lessens cytotoxicity with innate mucoadhesiveness and efficient penetration. The degree of quaternization and molecular weight are two essential parameters that elicit physicochemical and biological actions that support drug delivery approaches and biomedical applications of QCS (e.g., vaccine adjuvants) [193,194].

**Figure 5:**
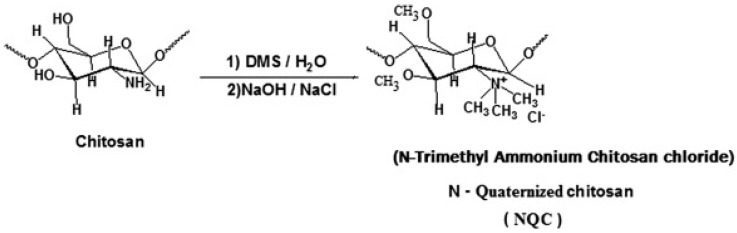
Synthesis of quaternized chitosan (DMS–dimethylsulfate) [198]. Reproduced with permission from Elsevier (Copyright © 2015).

N-[(2-hydroxyl-3-trimethyl ammonium) propyl] CS or N-substituted quaternized CS (HTCC) is the second-most-popular QCS prepared via an alkylation process involving the insertion of a quaternary ammonium group outside of the CS structure. Lang et al. (1980) synthesized the first HTCC through CS modification [195]. The reaction scheme of synthesis of quaternized CS is illustrated in Figure 5. The reagent, glycidyl trimethylammonium chloride (GTMAC), is often used for the direct replacement of hydrogen from attached amino and hydroxyl functional groups from CS at the C6 position to obtain O, HTCC, which enhances the aqueous solubility and antibacterial efficacy [196]. The generated HTCC polymers have different degrees of quaternization that relate to the extent of the positive charge [197].

##### Sulfated CS

The chemical surface of CS can be modified using sulfate groups, and their derivatives can produce polyampholytic chains similar to those found in the structure of sulfated glycosaminoglycans (GAGs), which represent a special class of charged polysaccharides involved in the formation of extracellular matrices, such as chondroitin sulfate and heparan sulfate [199]. Sulfated CS derivatives have a similar ability to bind with different growth factors and other proteins, enhancing or inhibiting the binding to their receptors. [200]. GAGs can interact with various proteins in the extracellular matrix, thus signaling to mediate various cellular processes, such as adhesion, migration, proliferation, and differentiation [201,202]. GAGs have numerous important pharmacological properties and biological activities, such as antiviral and anticoagulant activities, among others [203]. Therefore, GAG derivatives, such as sulfated CS (SCS), represent prominent candidates for use in several biomedical applications [204,205].

**Figure 6:**
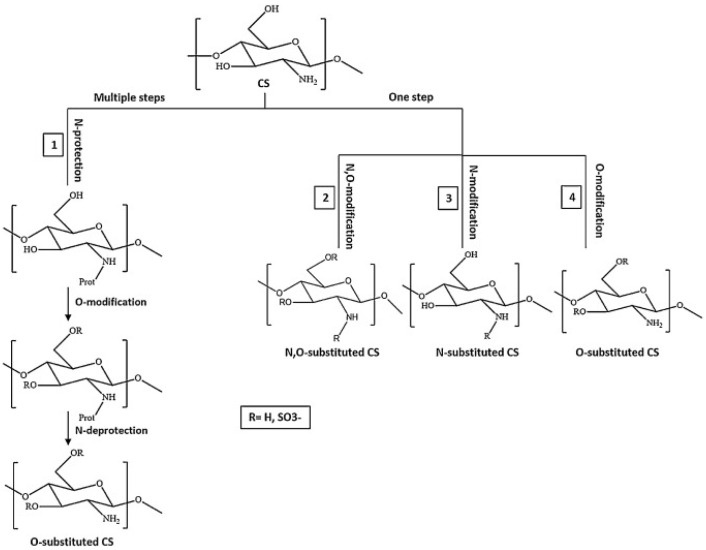
Synthesis of sulfated chitosan [217]. Reproduced with permission from Elsevier (Copyright © 2018).

CS sulfonation can occur at three key positions in the glucosamine and acetyl glucosamine residues: the C-2, C-3, and C-6 positions carrying the amino, secondary, and primary hydroxyl groups, respectively. This gives rise to N-, O- or N,O-sulfated CS, and, depending on the type of sulfonating reagent and reaction conditions, the reaction can lead to a selective or nonselective derivative. The synthesis of sulfonated CS is represented in Figure 6. Traditionally, CS sulfates have been prepared by using different sulfonating agents such as chlorosulfonic acid (HClSO_3_), 1-piperidinesulfonic, sulfuryl chloride, sulfuric acid, SO_3_, or sulfamic acid [206]. The modification of CS with sulfate groups can provide new or improved properties while retaining the original physicochemical and biochemical properties of CS, e.g., its low immunogenicity, biodegradability, and wound-healing activity [200]. SCS is a water-soluble anionic CS derivative, with a antiviral [207], anticoagulant [208], antimicrobial [209], and osteogenic activity [210,211]. This derivative also blocks human malignant melanoma cell adhesion [212] and shows an anti-obesity effect through the promotion of anti-adipogenesis inhibition [213]. In addition, sulfated CS has low cytotoxicity [208].

With specific regard to the antiviral effect, the material’s degree of sulfation (i.e., the number of sulfate groups per monosaccharide unit) is an important parameter for antiviral activity [214]. There is a positive correlation between the increasing degree of sulfation and antiviral potency [215]. In the cases of several human viruses, including HIV, it is thought that sulfated polysaccharides work through dual mechanisms: blocking viral adsorption and inhibiting viral replication [216].

##### CS Derivatives with Sugar Part

In particular, mono- and disaccharides, due to their availability and low cost, have been extensively used to obtain soluble branched CS derivatives. It is possible to summarize the most popular chemical modifications into three main approaches: (i) reductive N-alkylation, (ii) amide bond formation, and (iii) Maillard reaction. These reactions allow for obtaining a wide range of substitution degrees, depending not only on the operating conditions but also on the type of saccharide employed [218].

The use of reductive N-alkylation (i) for the covalent attachment of oligosaccharides to the primary amino functions of CS is a simple and versatile procedure. In 1984, Yalpani and Hall reported various N-alkylated CS derivatives, with various saccharides obtained by employing this approach [219]. Later, lactose-modified CS obtained similarly generated great interest in terms of biological applications due to its high biocompatibility and bioactivity [220].

The condensation (ii) between a carboxylic group on oligosaccharides and the free amino groups of CS is another method exploited to obtain glycosylated CS. Carbodiimide-mediated condensation has been largely employed to easily obtain soluble derivatives of CS [221,222]. Ruiz Matute et al. [223] used lactobionic acid to obtain biocompatible and bioactive modified CS. The condensation reaction has been exploited to obtain galactosylated derivatives able to interact with hepatocyte cells.

**Figure 7:**
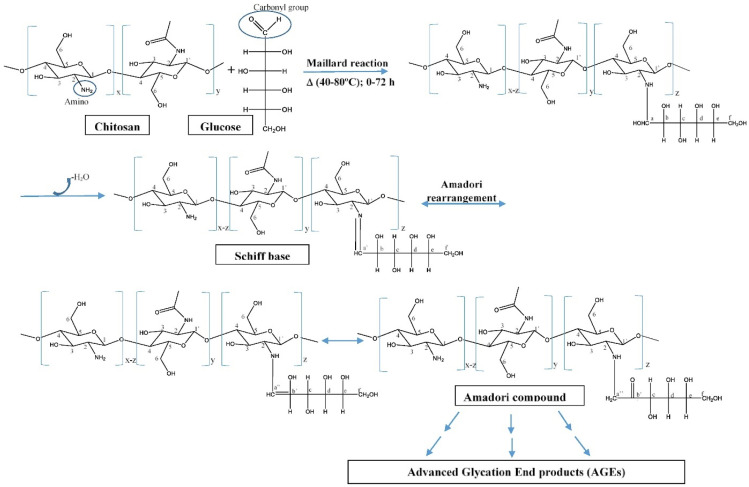
The reaction scheme for a CS derivative with sugar via the Maillard reaction [225]. Reproduced with permission from Elsevier (Copyright © 2016).

The Maillard reaction (iii), a form of non-enzymatic browning, is a chemical reaction involving the condensation between an amino group of proteins, amino acids, or any other nitrogenous compound and a carbonyl group of reducing sugars, i.e., aldehydes or ketones [224]. The presence of free amino groups makes CS a good candidate to react with reducing sugars through the Maillard reaction. The reaction scheme for a CS derivative with sugar via the Maillard reaction is illustrated in Figure 7.

Li et al. [226] synthesized a sialyl lactose-CS derivative by grafting a lactoside bearing an aldehyde-functionalized aglycone to the amino groups of CS, followed by enzymatic sialylation with sialyltransferase. The resulting glycosylate CS was found to be particularly appealing for binding the influenza virus surface hemagglutinin protein with high affinity, thereby inhibiting the viral attachment to host erythrocytes.

##### CS Glutaraldehyde Crosslinked Polymer

**Figure 8:**
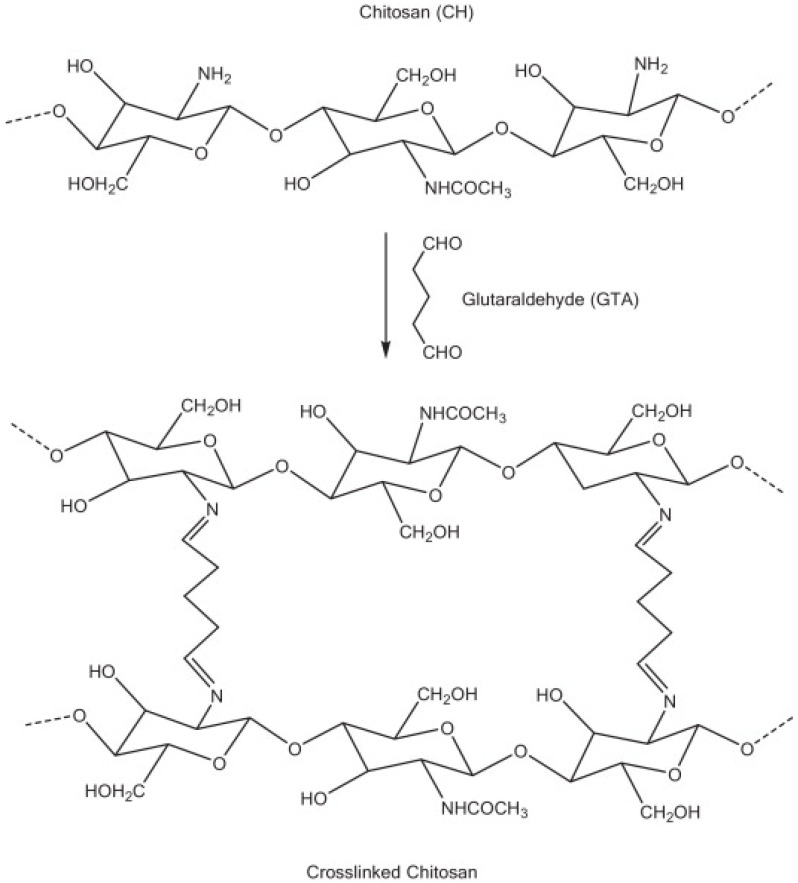
Crosslink reaction between chitosan and glutaraldehyde [228]. Reproduced with permission from Elsevier (Copyright © 2012).

The formation of CS crosslinked polymers through electrostatic complexation is based on the presence of cationic sites available all along the polymer chains (due to the protonation of amine groups in an acidic medium). The crosslink reaction between CS and glutaraldehyde is illustrated in Figure 8. When the protonation degree of these chains is sufficient, they can form complexes with anionic (macro)molecules to produce fine stable colloidal objects. Note that the “polyelectrolyte complexation” term is used when macromolecules (e.g., dextran sulfate, alginate, hyaluronic acid, DNA) are involved in nanoparticle formation. Meanwhile, when anionic species are used for the complexation of CS chains, that is, molecules such as citrate, sulfate, or (poly)phosphate, the method is more specifically termed “ionic gelation” [227].

Among the most commonly employed crosslinking agents are dialdehydes such as glyoxal and glutaraldehyde, a very reactive species capable of undergoing polymerization in an aqueous media [229]. Glutaraldehyde is the most widely used crosslinking agent due to its high stability despite its cytotoxicity [230,231].

Jain et al. [232] prepared CS microspheres using an emulsification technique based on glutaraldehyde crosslinking and loaded them with the antiviral model drug ACV. A variety of formulation and process variables such as the concentration of polymer, glutaraldehyde, drug, and Span, and the effect of stirring speed and stirring time. were optimized. The prepared microspheres showed a controlled drug release of 93.2% over 8 h with an initial burst release of 56.7% in the first 2 h. Fourier-transform infrared spectroscopy showed that there was no possible drug interaction between the drug and polymer. From the data obtained, it can be concluded that the CS microspheres could offer a possible carrier for controlled drug delivery of ACV.

##### CS Cyclodextrin

**Figure 9:**
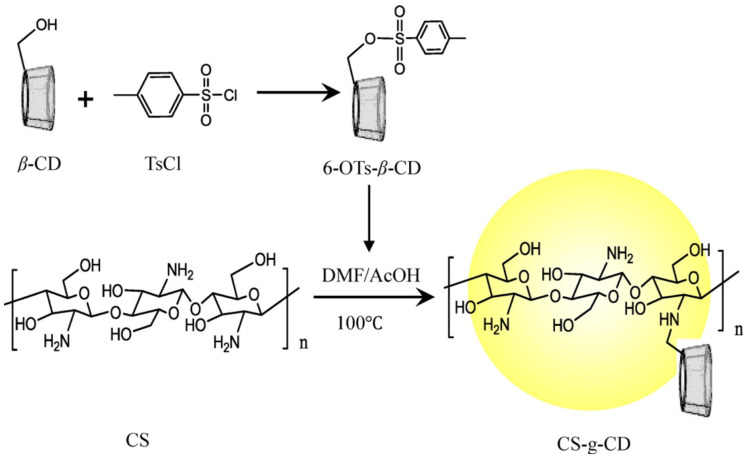
Reaction scheme for the synthesis of β-CD-CS derivative [236]. Reproduced with permission from Elsevier (Copyright © 2013).

Cyclodextrins (CDs) are a series of oligosaccharides produced by *Bacillus macerans* during the enzymatic degradation process of starch and related compounds. Among various CDs, β-cyclodextrin (β-CD), a natural molecule derived from starch, is a torus-shaped cyclic oligosaccharide [233]. As a result of electron-rich glycosidic oxygen atoms, the interior of the toroid or central cavity is hydrophobic. Consequently, hydrophobic molecules or groups can be included in cavities of β-CD in the presence of water [234]. Moreover, β-CD is known to enhance the solubility of the incorporated drug and to act as a permeation enhancer for different macromolecular drugs [235]. The synthesis of the β-CD-CS derivative is illustrated in Figure 9.

Malik et al. [237] evaluated the preparation of a pH-responsive hydrogel as an oral-controlled drug delivery system for ACV. Different proportions of β-CD, (CS), methacrylic acid, and N′N′-methylenebis-acrylamide were used to fabricate hydrogels via the free radical polymerization technique. The ACV release profile showed good fitting to zero-order kinetics, demonstrating a controlled release pattern. An acute oral toxicity study showed no significant changes in the behavior patterns of animals, biochemical blood analysis, and histopathology. A pharmacokinetic study revealed significant enhancement in the bioavailability of polymeric networks when compared with a marketed oral suspension. Considering the biocompatibility, pH-dependent swelling, and drug release behavior, it can be concluded that the developed hydrogel exhibited efficient drug carrier properties.

Donalisio et al. [238] created sulfobutyl ether-β-cyclodextrin decorated CS nanodroplets capable of incorporating and releasing ACV in a sustained manner.

##### CS Oligomers

**Figure 10:**
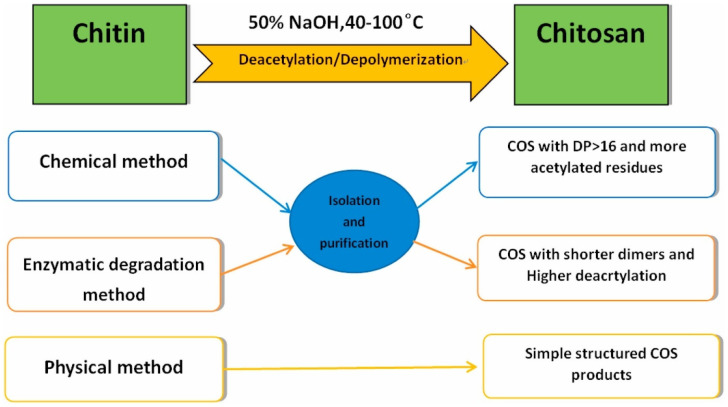
Preparation of COS from chitin and CS via physical, chemical, and enzymatic degradation methods [241]. Reproduced with permission from Elsevier (Copyright © 2021).

CS oligomers (CS oligosaccharides) (COS) can be considered low molecular derivatives of CS polymers (produced mostly by enzymatic cleavage). The preparation of COS from chitin and CS via physical, chemical, and enzymatic degradation methods is illustrated in Figure 10. COS systems possess enhanced intrinsic antiviral activity in comparison with their native polymeric counterparts. They also have better solubility than native polymeric systems. For this reason, they do not need derivatization. The main inhibition by COS is associated with its ability to inhibit the binding of viral proteins with the receptors of the host cells and also reduce the viral load through strong electrostatic repulsive interactions with the virus, COS being cationic [239]. COS also tend to stimulate the immune response of healthy cells against foreign organisms such as viruses. Lastly, CS and its derivatives also act as good, selective, and efficient carriers for antiviral drugs. COS is more soluble in an acidic aqueous medium in comparison with high-molecular-weight CS, but its antimicrobial activity decreases with an increase in the acetylation degree and an increase in pH above the critical pH point for a CS solution. COS, composed of a few monomer units of CS derived from CS degradation, shows greater inhibition toward neuraminidase activity in vitro than hetero-oligosaccharides of lower molecular weight and lower degrees of acetylation [240].

COS are water soluble and can offer a potential drug carrier for renal-targeted delivery. Zidovudine (AZT), an FDA-approved antiretroviral drug, has a very short half-life and is eliminated very quickly in human plasma and kidneys after administration. In the study by Liang et al. [242], AZT–COS conjugates were prepared and evaluated in terms of renal targeting. The in vitro release of AZT from AZT–COS conjugates was confirmed in mice plasma and renal homogenate. The pharmacokinetics study indicated a longer mean retention time of AZT–COS conjugates with values of about 1.5 h than 0.59 h of AZT. The AZT–COS conjugates were found to be accumulated in the kidneys more so than the heart, liver, spleen, lung, and brain after IV administration, in line with evidence from the fluorescence imaging of FITC-labeled COS after 12 h. In conclusion, AZT–COS conjugates have the potential to be developed into a renal-targeting drug delivery system.

Klausner et al. [243] designed and characterized CS-DNA NPs based on NOVAFECT. NOVAFECT chitosans are ultrapure CS oligomers that were recently marketed as carriers for non-viral gene therapy. In rat corneas, injection of a select formulation of oligomeric CS-DNA NPs into the stroma showed that (i) luciferase gene expression was 5.4 times greater than following administration of polyethylenimine-DNA NPs; and (ii) the cells that expressed the transgene green fluorescent protein were keratocytes (corneal fibroblasts). Their study lays the foundation for evaluating chitosan oligomers as carriers in corneal gene therapy, ocular gene therapy, and various additional gene therapy applications.

### 4.2. CS and Its Derivatives in Nanoparticulate Antivirotic Drug Delivery Systems

#### 4.2.1. Properties and Activities of CS-Based Nanoparticulate Systems

Polymeric nanosystems have unique physicochemical characteristics such as (i) prolonged blood circulation time; (ii) reduced adverse effects; (iii) ability to protect therapeutic agents from degradation, increasing the stability, bioavailability, and pharmacokinetics of drugs; (iv) ease of chemical modification; (v) controlled drug release; and (vi) improved therapeutic effects [22]. Nanocarriers also offer the advantages of improved drug solubility, higher loading capacity, and efficiency of drug payloads. The high surface area of the polymeric NPs allows for a highly efficient drug carrier, and the size becomes a determining characteristic at the moment of interaction with the cell membrane and in the penetration of physiological barriers [244].

CS-based NP systems confer desirable attributes of biodegradability, biocompatibility, permeation enhancement, mucoadhesiveness, and antimicrobial and antifungal activity, along with extremely useful efflux inhibitor properties. CS provides positive zeta potential, offering better control of the physicochemical properties, changes in protein corona formation, and enhanced cellular uptake, resulting in improved drug efficacy. Although there is an urgent need to examine its side effects and toxicity, in the near future, there is a huge untapped, emerging potential for CS NPs as advanced delivery systems in biomedical and pharmaceutical fields. As an example of effectivity in drug delivery and enhancement of the therapeutic efficacy of the drugs, CS NPs are advantageously used in ocular drug delivery due to CS in situ gelling properties and mucoadhesive character. They are also used in oral drug delivery as they open the tight junctions of the mucosal membrane and enhance absorption. Moreover, their positive charge makes them helpful in pulmonary drug delivery. Elsewhere, they increase the permeability of various drugs, making them available for nasal delivery. Furthermore, they enhance the absorption of hydrophilic molecules, thereby helping with mucosal drug delivery [15].

CS acts as an adjuvant in CS NP-based vaccines delivered via mucosal and systemic routes and is particularly useful in mucosal delivery (because it promotes absorption and mucosal immune response) and in systemic administration. It is protected from enzymatic degradation in the mucosal tissue and facilitates antigen uptake by mucosal lymphoid tissue; thus, DNA mucosal vaccines are widely explored. It also demonstrates the potential for stimulating humoral and cellular immune responses [23]. Poecheim et al. [194] investigated the stability of cationic NPs made of N-trimethyl chitosan and chondroitin sulfate (TMC NPs) in an aqueous solution and after freeze-drying. Furthermore, the structural integrity of plasmid DNA (pDNA) on adsorption to the nanoparticle surface was investigated. Unloaded TMC NPs remained stable in suspension for 24 h, maintaining diameters of around 200 nm and zeta potential values of approximately 38 mV. Freeze-drying with sucrose could ensure storage for 30 days, with minimal increase in size (291 nm) and charge (62 mV). Results showed that TMC NPs may potentially be freeze-dried in the presence of sucrose to be stored for prolonged periods. In addition, pDNA was successfully adsorbed to the cationic NPs and remained intact after being released.

In recent years, nanotechnology has been applied to increase the effectiveness of CS as an antiviral agent. CS NPs have improved physical characteristics and can be used as carrier molecules to enhance the antiviral activity of certain antiviral agents [23].

**Figure 11:**
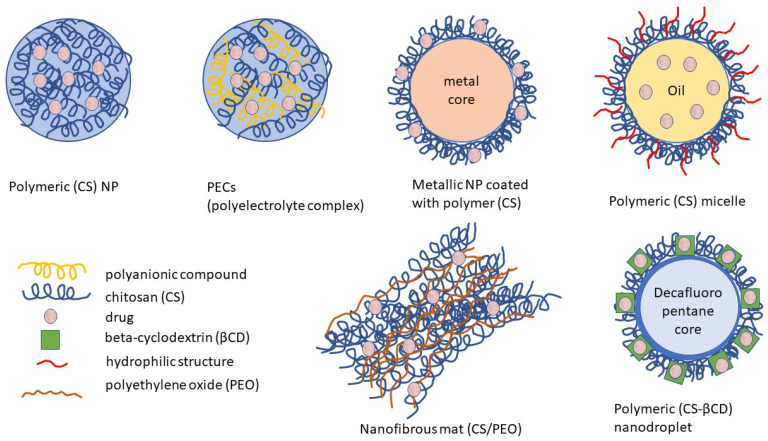
Different types of CS NPs in antiviral drug delivery systems.

The structure of CS NPs used in antiviral drug delivery systems is illustrated in Figure 11 and a list of CS NPs containing antiviral drugs, with a critical evaluation of their benefits and limitations for potential antiviral applications, is presented in Table 3, and. Mechanisms of action of particular CS NP-based drug delivery systems, along with a degree of their implementation in practice, are briefly mentioned, too. The CS-based antiviral nanocarriers are discussed concerning their preparation and antiviral delivery examples in Section 4.2.2 and Section 4.2.3, respectively.

#### 4.2.2. Preparations of CS-Based Nanoparticulate Systems

**Figure 12:**
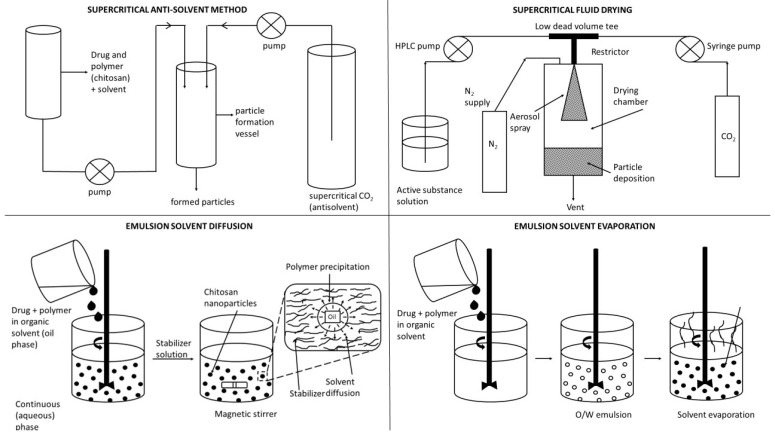
Schematic representation of preparation methods of CS NPs applied for antivirals.

PNPs (polymer nanoparticles) can be conveniently prepared either from preformed polymers (via polymer dispersion) or by direct polymerization of monomers using classical polymerization or polyreactions. The choice of preparation method is based on several factors such as the type of polymeric system, area of application, size requirement, etc. There are several methods for CS NP preparation. The most frequent are the following: (i) solvent evaporation method, (ii) spontaneous emulsification/solvent diffusion method, (iii) production of NPs using supercritical fluid, and (iv) supercritical anti-solvent (SAS) method [262]. These CS NP preparation methods were used for the CS nanocarriers of antivirotics summarized in Table 3 and discussed in more detail in Section 4.2.3. The methodological principles applied therein are briefly described in the following paragraphs (i)–(iv), and the schemes of preparation are illustrated in Figure 12.

(i) Solvent evaporation method: In this method, the polymer is dissolved in an organic solvent such as dichloromethane, chloroform, or ethyl acetate. The drug is dissolved or dispersed into the preformed polymer solution, and this mixture is then emulsified into an aqueous solution to make an O/W emulsion by using a surfactant/emulsifying agent such as gelatin, poly(vinyl alcohol), polysorbate-80, poloxamer-188, etc. After the formation of a stable emulsion, the organic solvent is evaporated by increasing the temperature, raising the pressure, or continuously stirring. The W/O/W method has also been used to prepare the water-soluble drug-loaded NPs. Both the above methods use high-speed homogenization or sonication. However, these procedures are good for a laboratory scale only [263].
(ii) Spontaneous emulsification/solvent diffusion method: The water-soluble solvent such as acetone or methanol along with the water-insoluble organic solvent such as dichloromethane or chloroform are used in an oil phase. Due to the spontaneous diffusion of water-soluble solvent (acetone or methanol), interfacial turbulence is created between two phases, leading to the formation of smaller particles. As the concentration of water-soluble solvent (acetone) increases, a considerable decrease in particle size can be achieved [9,264].
(iii) Production of NPs using supercritical fluid: Supercritical fluids have now become attractive alternatives because these are environment-friendly solvents and the method can be profitably used to process particles at a high purity and without any trace amount of the organic solvent. Rapid expansion of supercritical solution (RESS) method is a method where the solute of interest is solubilized in a supercritical fluid and the solution is expanded through a nozzle. Thus, the solvent power of supercritical fluid dramatically decreases and the solute eventually precipitates. This technique is clean because the precipitated solute is completely solvent-free. Unfortunately, most polymers exhibit little or no solubility in supercritical fluids, thus leaving the technique of little practical interest [16].
(iv) Supercritical anti-solvent (SAS) method: The solution is charged with the supercritical fluid in the precipitation vessel containing a solute of interest in an organic solvent. At high pressures, enough anti-solvent enters the liquid phase that the solvent power is lowered and the solute precipitates. After precipitation, when the final operating pressure is reached, the anti-solvent flows through the vessel to strip the residual solvent. When the solvent content has been reduced to the desired level, the vessel is depressurized and the solid product is collected. In a modified version of the SAS technique, the solid of interest is first dissolved in a suitable solvent and then this solution is rapidly introduced into the supercritical fluid through a narrow nozzle. The supercritical fluid completely extracts the solvent, causing the supercritical fluid insoluble solid to precipitate as fine particles. This method, also called the gas anti-solvent (GAS) technique, has been successfully used to produce micro-particles as well as NPs [248].

#### 4.2.3. Applications of CS-Based Nanoparticulate Systems in Antiviral Drug Delivery

**Figure 13:**
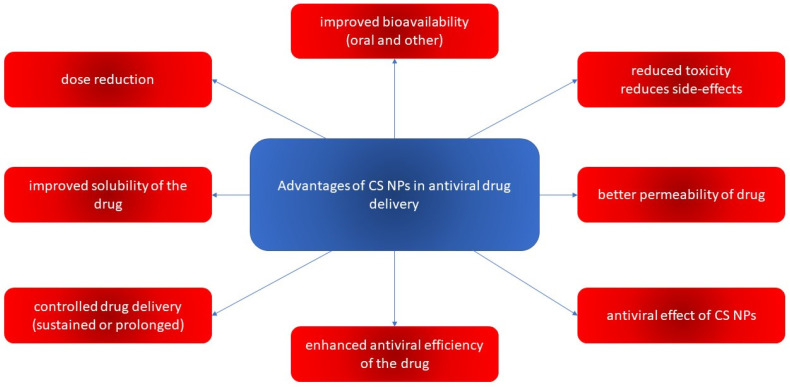
Main advantages of CS NPs in antiviral drug delivery systems.

Recent in vitro and in vivo applications of CS NPs and CS nanocomposites in the progressive delivery of antivirotics are briefly discussed in this section, highlighting CS’s role in such nanoparticulate systems in italics at the beginning of each relevant paragraph. Table 3 represents the list of corresponding CS-based nanosystems providing various antiviral drugs’ improved attributes, such as stability, selectivity, bioavailability, physicochemical properties, solubility, and modified drug release, and their different administration routes. Figure 13 illustrates the main advantages of CS NPs in antiviral drug delivery systems.

##### Nanoparticles with Native CS

*Bioavailability’s and physicochemical properties’ improvement of silymarin.* To develop a specific treatment against COVID-19, Loutfy et al. [257] investigated silymarin–CS NPs (Sil–CNPs) as an antiviral agent against SARS-CoV-2 using in silico and in vitro approaches. Docking of Sil (silymarin) and CNPs was carried out against SARS-CoV-2 spike protein using AutoDock Vina. CNPs and Sil–CNPs were prepared by ionic gelation. An in silico study showed high binding energies of the tested agents, especially SARS-CoV-2. CNP proved to be a promising antiviral agent against ADV-5 (adenovirus type 5) and SARS-CoV-2 in vitro and its conjugation to Sil improved its bioavailability and physicochemical properties. The increased antiviral activity might be achieved via blocking viral host receptor ACE2, thus preventing viral attachment and its entry into the cells. These results may need further investigations to confirm blockage of the receptor during viral infection and to build the metabolic profile, before proceeding to an in vivo study, probably in the form of intranasal drug delivery to inhibit viral entry.

**Figure 14:**
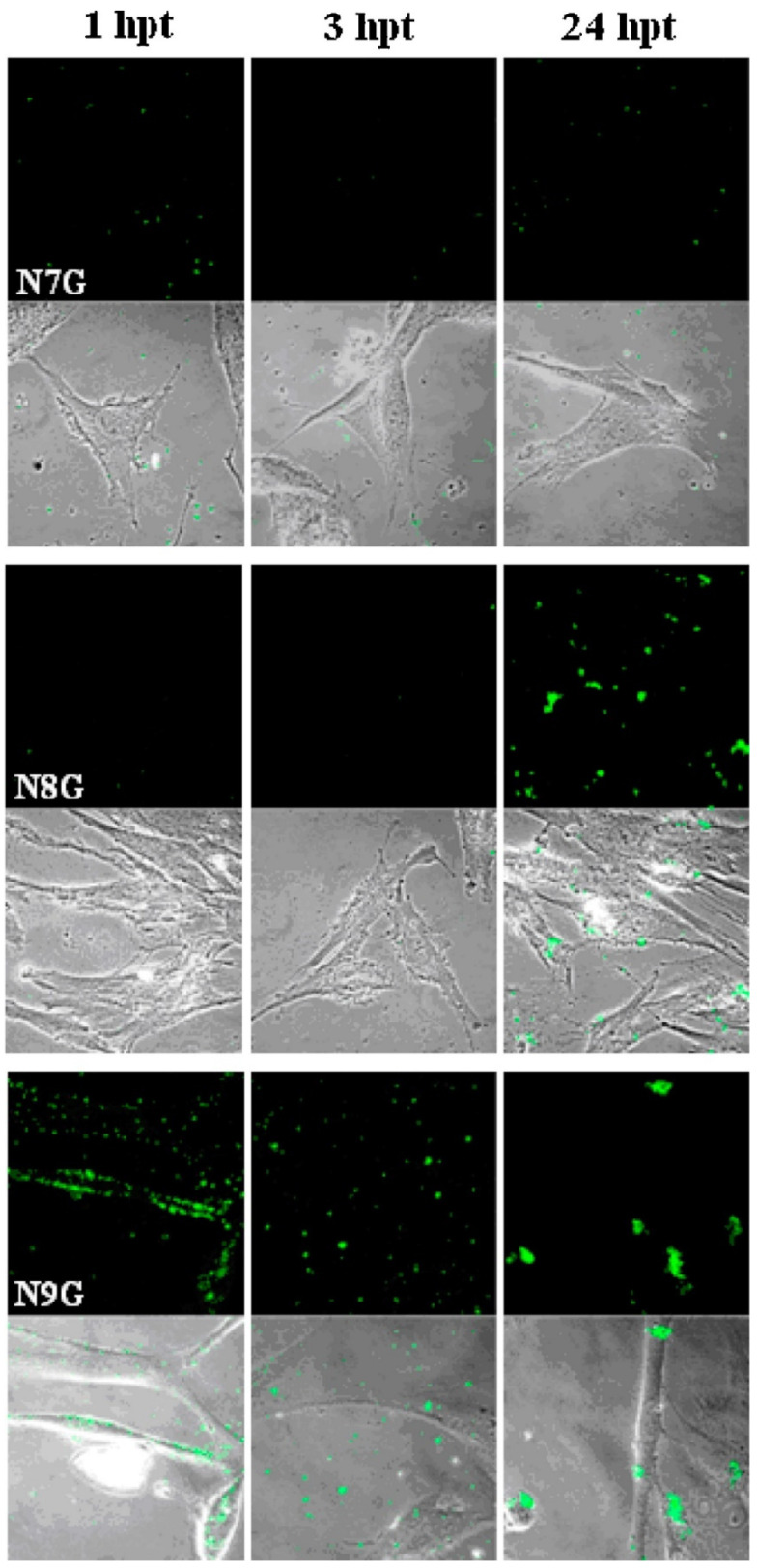
Evaluation of cellular uptake of fluorescent N7G, N8G, and N9G samples (CS NPs loaded with foscarnet). HELF cells (human embryonic lung fibroblasts) were incubated with the formulation for the periods indicated and then analyzed by confocal laser scanning microscopy without fixation at 1, 3, and 24 h post-treatment (hpt). The upper panels show fluorescent images while the lower panels show fluorescent images merged with phase-contrast images [256]. Reproduced with permission from Elsevier (Copyright © 2014).

*Prolonged release and absorption increase of foscarnet.* A major problem associated with the administration of foscarnet is its marked toxicity. The major adverse effect of foscarnet is renal dysfunction, which appears to be less severe and less prevalent with intermittent dosing than with continuous infusion. Another frequent side effect is related to fluctuations in serum calcium levels, with both increased and decreased serum calcium levels. Acute hypocalcemia during the infusion of foscarnet may lead to muscular spasms, paresthesias, tremors, arrhythmias, and seizures, which are reversed if the rate of infusion or the drug dosage is reduced [265,266]. Russo et al. [256] encapsulated foscarnet into potentially long-circulating CS NPs, which led to a prolonged blood residence and in vivo exposure of infected cells to the drug, accompanied by reduced toxic effects. Meanwhile, absorption was increased by the mucosal epithelium due to the CS mucoadhesive properties, which could also contribute to making both the oral and topical routes more effective for the administration of foscarnet. An evaluation of the cellular uptake of the prepared foscarnet-loaded CS NPs by HELF cells (human embryonic lung fibroblasts) is shown in Figure 14.

*Stability and protein packing efficiency enhancement for improvement of oral bioavailability and gastroresistance of IFNα.* CS NPs have high stability and high protein packing efficiency. They can also be used as advanced drug delivery systems due to their capacity to alter protein loading. IFNα has a very short half-life in vivo (2–3 h IM/SC; 2 h IV) (intramuscular/subcutaneous) and a narrow therapeutic index. A pegylated form was developed to prolong its half-life and enable a once-a-week injectable administration. However, PEG-IFNα injections cause pain, allergic reactions, and poor patient compliance [267,268]. Cánepa et al. [252] hypothesized that the encapsulation of IFNα within CS NPs would protect the drug from gastric degradation and improve its oral bioavailability compared to the free counterpart. An additional advantageous feature of CS stems from its capacity to increase the uptake of macromolecules by opening the tight junctions in the intestinal epithelium. They described the design and full characterization of a novel CS-based nanocarrier for the oral delivery of IFNα-2b, aiming to replace its parenteral administration. The synthesis of the IFN-loaded CS NPs is simple, reproducible, and eventually scalable, and the %EE (encapsulation efficiency) is almost 100%. The encapsulated drug conserved its antiviral activity, a remarkable advantage over pegylated counterparts that present sharp activity loss. Finally, in a preliminary pharmacokinetic study, they demonstrated that these NPs enhance the oral absorption of IFNα, which could be detected in plasma, as opposed to the free derivative, which was undetectable.

*Permeation and bioavailability enhancement and systemic toxicity reduction* of nevirapine. Nevirapine (NVP)-loaded CS NPs were prepared via the salting out method, and 3^2^ randomized factorial designs were used to optimize the formulation. The results of a FTIR (Fourier-transform infrared spectroscopy) and DSC (differential scanning calorimetry) study confirmed the absence of incompatibility of NVP with excipients used in the formulations. A stable nanoformulation was developed successfully with very good permeation of the drug across vaginal tissue, which may increase the bioavailability in the target area and is, therefore, expected to increase the therapeutic effect of NVP and reduce the systemic toxicity. Further in vivo pharmacokinetics and pharmacodynamic studies, along with safety and efficacy assessments, are required in the future to prove the value of the proposed nanocarriers [261].

*Bioavailability enhancement of hydrophobic drugs* (saquinavir, dolutegravir). A poor water solubility and intrinsic dissolving rate are the key difficulties influencing the oral distribution of many existing drugs, such as antivirals for HIV4. Conventional saquinavir therapy is not sufficiently effective due to its poor bioavailability [269]. The poor bioavailability of saquinavir is mainly attributed to a group of MDR1 (multi-drug-resistant 1) proteins along with the P-gp mediated efflux system, which causes the elimination of the drug due to its resemblance with the substrate analog of the P-gp mediated system [270,271]. In a recent paper, Ramana et al. [272] employed CS nanocarriers loaded with saquinavir to overcome the limitations of antiretroviral therapy, which is currently the most effective treatment for AIDS. The results showed a loading efficiency of 75% and a cell targeting efficiency of more than 92%, as well as a swelling percentage of CS at various pH levels. These findings revealed that a combination of diffusion and erosion accounts for saquinavir release from the CS matrix. The rate of drug release is proportional to the amount of drug contained in the matrix, according to first-order release kinetics. The erosion mechanisms are more prevalent at alkaline pHs, resulting in a progressive change in the surface area, according to the saquinavir release profiles.

To enhance the solubility and improve dolutegravir (DTG) bioavailability, CS NPs were synthesized utilizing spray-drying technology. The developed nanoformulation was characterized for its physicochemical properties and investigated for the feasibility of its administration through an oral route along with milk/food as an admixture for pediatric antiretroviral therapy. In vivo oral bioavailability studies were conducted in Balb-C mice, where the animals were treated with the selected formulation of DTG-loaded CS NPs and compared to pure DTG. The findings showed that CS-based NPs were ideal carriers for oral administration of DTG along with milk and exhibited great potential to enhance the bioavailability of the drug and improve treatment adherence for pediatric HIV patients [273].

##### Nanosystems Based on Polymeric Associates with Native CS (PECs, NFs)

*Controlled release of zidovudine.* To prepare a controlled-release system for zidovudine (AZT), Yan et al. [254] selected curdlan sulfate (CRDS) with a flexible random coil chain as a polyanion to construct nanosized polyelectrolyte complexes (PECs) with a polycation CS via electrostatic interactions. AZT as a model antiviral drug was successfully encapsulated in the nanosized PECs with a favorable drug-loading efficiency. AZT-loaded PECs were successfully formulated, and the in vitro drug release at various pH levels was studied and evaluated. The results implied that the fabricated CRDS/CS PECs could be explored as potential nanocarriers for delivering antiviral drugs such as AZT for controlled release.

**Figure 15:**
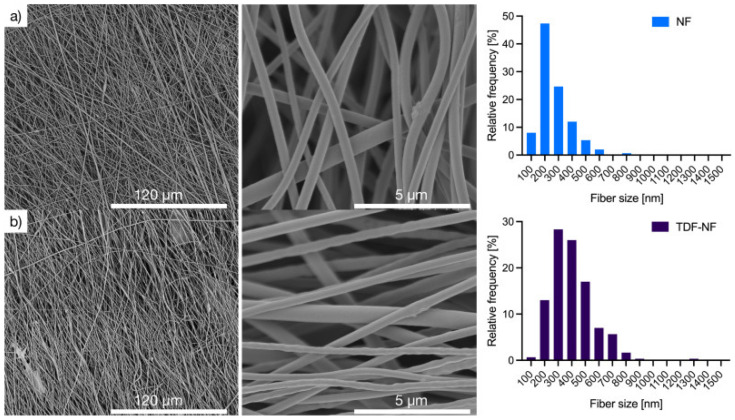
Representative SEM (scanning electron microscope) images and fiber size distributions (*n* = 300) of (**a**) drug-free nanofibrous mat (NF) and (**b**) tenofovir disoproxil fumarate-loaded nanofibrous mat (TDF-NF) prepared with CS and PEO at a ratio of 1:4 (*w*/*w*). SEM images magnifications: ×1000 and ×10,000 [258]. Reproduced with permission from Elsevier (Copyright © 2022).

*Prolonged release and permeation increase of tenofovir disoproxil fumarate*. Viruses including herpes simplex virus (HSV), human immunodeficiency virus (HIV), hepatitis B (HBV), and human papillomavirus (HPV) are responsible for incurable infections among pathogens linked to the incidence of sexually transmitted infections [274]. One of the main prophylactic strategies relates to developing pharmaceutical vehicles capable of enhancing microbicide performance for better protection from sexually transmitted infections [275]. Szymańska et al. [258] discovered that fluctuations in the physiological pH range of 3.8–5.0 substantially affected mucoadhesive behavior and the drug dissolution rate from the nanofibrous (NF) carrier. A more acidic vaginal environment favored nanofibrous mat adherence to the human vaginal tissue and sped up drug diffusion from the polymer matrix, which, in turn, may have increased the drug residence time with mucosal tissue and helped to initiate microbicide absorption onset. In turn, moderate prolonged drug release from the CS/PEO (CS-polyethylene oxide) nanofibrous mat, achieved in simulated vaginal fluid with pH 5.0, may have compensated for more pronounced tenofovir disoproxil fumarate (TDF) permeation across the vaginal epithelium. Representative SEM images of TDF/FTC-NPs are shown in Figure 15.

##### CS–Metal Nanocomposites

*Stability and selectivity enhancement of metal NPs.* Silver nanoparticles (AgNPs) and nanocomposites have been demonstrated to possess inhibitory properties against several pathogenic microbes, including archaea, bacteria, fungi, algae, and viruses [276]. AgNPs have been incorporated into nanocomposites to enhance stability [277]. Mori et al. [247] synthesized AgNP/CS composites with antiviral activity against influenza A virus in an aqueous medium. For all sizes of AgNPs tested, the antiviral activity of the AgNPs/CS composites increased as the amount of AgNPs increased. Stronger antiviral activity was generally observed with composites containing smaller AgNPs for comparable concentrations of AgNPs. Neat CS did not exhibit antiviral activity, suggesting that AgNPs are essential for the antiviral activity of composites. In another work focused on combinations of AgNP with CS, Sofy et al. [248] report the synthesis of poly quaternary phosphonium-based oligoCS (PQPOC). PQPOCs were used as synergistic reductants for Ag(I) in preparation of AgNPs and capping agent to stabilize them through the fabrication of PQPOCs-AgNPs nano-biocomposites. The enhanced antiviral performance of nano-biocomposites could be attributed to multiple additive effects, such as: (i) AgNPs could interact with the virions’ active sites (such as glycoproteins) and prevent viral attachment and penetration. (ii) PQPOC served as a viral interior inhibitor by blocking the interaction of targeted viruses with the host due to the electrostatic interaction between the positive brushes of PQPOC and negatively charged binding sites of viruses. (iii) The CS skeleton in PQPOC could induce ribonuclease to degrade the viral RNA and consequently prevent its transcription and translation. Both studies initiate a promising strategy for the preparation of TPP-based CS/AgNP composites, which may lead to future integrated antiviral research.

**Figure 16:**
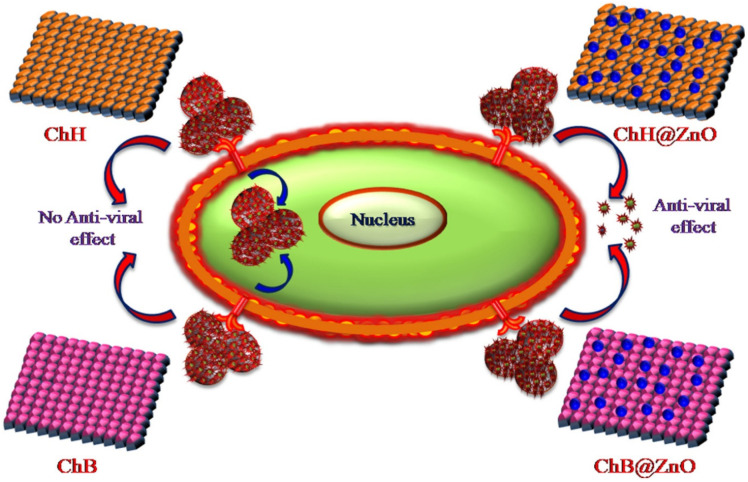
Probable mechanism of antiviral activity by the nanocomposites [245]. Reproduced with permission from Elsevier (Copyright © 2022).

Another metal-based nanocomposite is a combination of zinc oxide nanoparticles and CS (CS/ZnO NPs). CS/ZnO NPs are stable and potentially have antimicrobial activities. They have a strong inhibitory effect on HSV-1 and H1N1 influenza virus. Jana et al. [245] synthesized two types of composites, namely ChB@ZnO (CS/4-(benzyloxy)benzaldehyde) and ChH@ZnO (CS/4-hydroxybenzaldehyde/ZnO). ChB@ZnO and ChH@ZnO composites showed improved photophysical stability compared to bare ZnO NPs prepared in a hydrothermal process. ZnO NPs embedded in phenyloxy-functionalized CS composites have active antiviral potential against human cytomegalovirus in vitro with minimal cytotoxicity and can be effectively used either singularly or in combination with other anti-human cytomegalovirus (HCMV) drugs. It was observed that ChB@ZnO and ChH@ZnO composites largely decrease the viral load within the cells at around 72 hpi, suggesting that these materials mostly interfere with the active replication of the virus, thereby decreasing its copy number. The antiviral activity of the materials is possibly caused by ZnO micro-nanostructures’ inhibitory effect on HCMV through direct interaction with virus particles, which deceive the virions and, consequently, block the entry of HCMV into target cells, as is depicted in Figure 16.

##### CS–Selenium Nanocomposites

*Antioxidant capacity enhancement of selenium NPs.* Porcine reproductive and respiratory syndrome virus (PRRSV) is a highly prevalent and endemic swine pathogen that causes significant economic losses to the global swine industry. Selenium nanoparticles (SeNPs) have attracted increasing attention in the biomedical field given their antiviral effects. The study by Shao et al. [259] demonstrated that CS-coated SeNPs synthesized by chemical reduction could enhance the antioxidant capacity and effectively suppress PRRSV-induced apoptosis in Marc-145 cells via the ROS/JNK signaling pathway, thereby inhibiting PRRSV replication. PRRSV can block cellular antiviral defense mechanisms and hijack host apoptosis for dissemination and infection. Growing evidence corroborates the notion that PRRSV can suppress host apoptosis at an early stage of infection by activating the PI3K/AKT pathway and inhibiting the pro-apoptotic protein Bad.

##### CS–Graphene Oxide Nanocomposites

**Figure 17:**
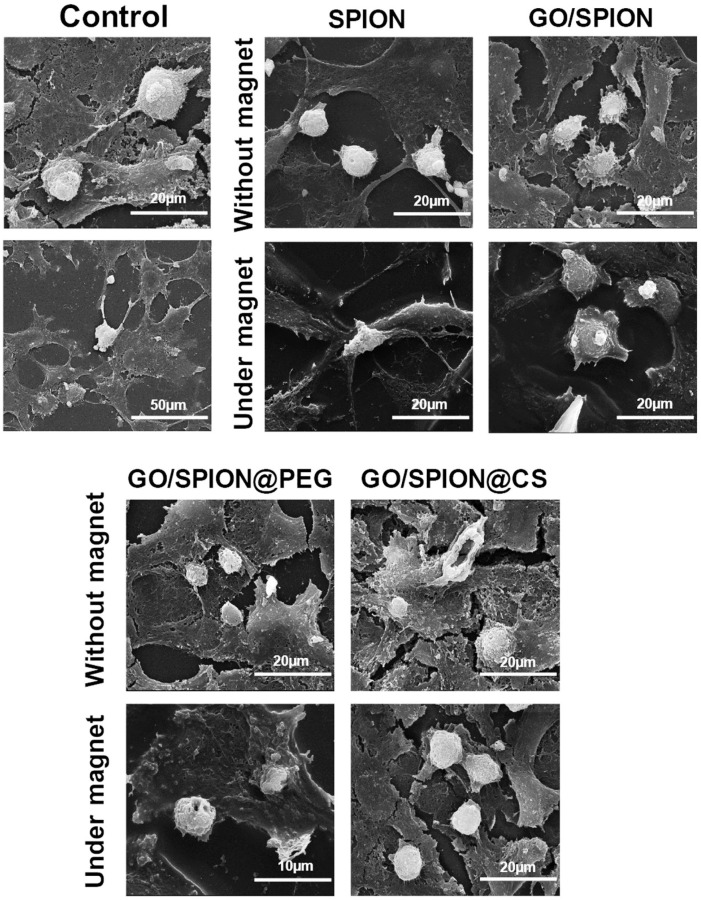
FESEM (field emission scanning electron microscopy) images of cell morphology of L929 cells exposed to the SPION NPs as well as GO/SPION nanocomposites in the presence or absence of a constant magnet [255]. Reproduced with permission from Elsevier (Copyright © 2022).

*Antiviral enhancement due to GO and CS synergism.* Graphene oxide (GO) is the oxidized form of graphene with hydroxyl, epoxide, diol, ketone, or carboxyl functional groups located on its surface. The presence of oxygen on the edges and basal planes of GO increases its hydrophilicity, water dispersibility, and attachability in comparison with graphene [278,279]. GO can exhibit outstanding properties such as a large specific surface area, high mechanical strength, and high electron conductivity, as well as strong thermal, optical, and catalytic characteristics. These unique properties of graphene and its derivatives make these nanostructured materials suitable for various biological and medical applications including anti-pathogenic applications. The progressive and fatal incidence/outbreak of some diseases such as cancer and coronavirus necessitate using advanced materials to bring such devastating illnesses under control. Kohzadi et al. [255] synthesized graphene oxide (GO) decorated by superparamagnetic iron oxide nanoparticles (SPION) (GO/SPIONs) as well as polyethylene glycol functionalized SPION (GO/SPIONs@PEG), and CS functionalized SPION (GO/SPIONs@CS). PEG and CS functionalized SPIONs were uniformly decorated on GO sheets. GO sheets decorated with SPIONs as well as GO/SPIONs@PEG and GO/SPIONs@CS demonstrated a super-paramagnetic behavior. FESEM (field emission scanning electron microscopy) images of the cell morphology of L929 cells exposed to the SPION NPs, as well as GO/SPION nanocomposites in the presence and absence of a constant magnet, are illustrated in Figure 17. Due to the polymer coating, *M _s_* values of functionalized GO/SPIONs were lower than those of GO/SPIONs but not pronounced for GO/SPIONs@PEG. The developed GO/SPION demonstrated low toxicity on L929 cells even up to 500 ppm of concentration. The highest level of SARS-CoV-2 virus inhibition was for GO/SPION@CS (86%) due to the synergistic exploitation of GO and CS. Regarding the effects of GO and hybrid GO/SPIONs on cell viability, the mechanism is not well explained and still requires further analysis.

##### Intrinsic Antiviral Activity of Derivatized CS NPs

Some of the CS derivative-based NPs (similar to their counterparts’ polymer systems) show antiviral activity themselves. TMTCS (trimethylCS) also possesses antiviral activity [280]. Milewska et al. [249] demonstrated the broad-spectrum antiviral activity of the cationically modified CS-HTCS (N-(2-hydroxypropyl)-3-trimethylammonium CS) NPs, which inhibited all low-pathogenicity human coronaviruses (HCoV-NL63, HCoV-229E, HCoV-OC43, and HCoV-HKU1). Using in vitro and ex vivo models of human airway epithelia, they showed that HTCS effectively blocks MERS-CoV and SARS-CoV-2 infection. The polymer blocks the virus entry into the host cell by interacting with the S protein.

##### Nanoparticles Based on Cyclodextrin Derivatives and Composites with Native CS

**Figure 18:**
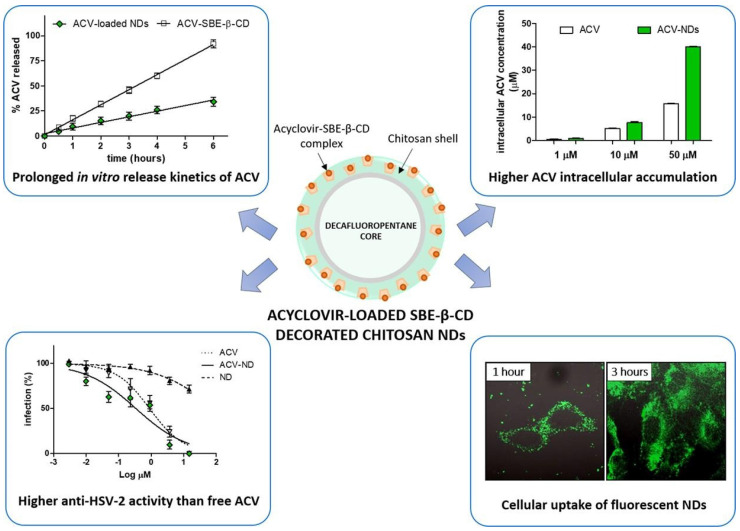
Properties of ACV-loaded SBE-β-CD (sulfobutyl ether-β-cyclodextrin)-decorated chitosan nanodroplets (NDs) [238]. Reproduced with permission from Elsevier (Copyright © 2020).

Sustained release, permeability, and activity enhancement of hydrophobic drugs (acyclovir, efavirenz) and proteins can be achieved through cyclodextrin/CS topical delivery. However, vaginal topical therapy with ACV is hampered due to its poor bioavailability and low retention at the vaginal mucosa, thus requiring high doses and frequent administrations. Donalisio et al. [238] focused on developing a novel formulation consisting of sulfobutyl ether (SBE)-β-cyclodextrin-decorated nanodroplets for ACV topical delivery to improve their antiviral effectiveness in topical vaginal application. The structure and properties of ACV-loaded SBE-β-CD are illustrated in Figure 18. ACV was efficiently incorporated in the nanodroplets (about 97% encapsulation efficiency) and they slowly released ACV in a sustained manner. In addition, they exhibited enhanced antiviral activity compared to the free drug against HSV-2 in cell cultures, which might be ascribed to a higher intracellular accumulation of the drug in nanodroplet-treated cells than in free ACV-treated cells. In another work, Donalisio et al. [281] loaded ACV in a CS NP composite with cyclodextrin for the treatment of HSV-1 and HSV-2. These NPs successfully improved the topical delivery of ACV through the skin by increasing its permeability compared to its free form. In vitro studies showed higher antiviral activity of ACV-loaded NPs against both HSV strains.

The incompetence of antiretrovirals (ARV) in the complete eradication of HIV from the CNS is the biggest issue in neuro-AIDS treatment. The ineffectiveness is largely due to the poor penetration of antiretrovirals. A factor that limits the use of CS in sustained-release applications is its hydrophilicity, resulting in a poor capacity to carry hydrophobic molecules. Efforts have been made to improve the ability of CS to carry hydrophobic substances. In some studies, the advantages of both carriers (mainly CS with cyclodextrin) have been combined in order to develop more efficient carrier systems, such as those providing improved mucoadhesive properties for the effective release of drugs for biomedical purposes [282].

Belgamwar et al. [283] described how to enhance the CNS uptake of efavirenz by designing intranasal efavirenz NPs (EFV-NPs). EFV-NPs were fabricated using CS/g-HPβCD (CS-graft-2-hydroxypropyl-β-cyclodextrin) and the ionic gelation method and optimized using a quadratic response surface methodology employing a two-factor, five-level circumscribed central composite design. Low-sized, stable NPs with high drug loading could be successfully prepared using CS-g-HPβCD. Sustained release from the NPs and enhanced permeation through the nasal mucosa of EFV could be achieved without adverse effects. Significantly greater access of EFV to the CNS and a high drug targeting index to the brain from intranasal EFV-loaded CS-g-HPβCD NPs makes this a promising route for targeting the CNS in Neuro-AIDS.

##### COS Nanosystems

Despite the aforementioned advantages of CS, its poor aqueous solubility at a physiological pH is considered the major limitation (readily soluble in acidic medium only). The merits of COS such as its high water solubility, low viscosity, biocompatibility, biodegradability, mucoadhesiveness, and permeation enhancing capability boost its potential applications in pharmaceutical and biomedical fields [284,285].

*pH sensitivity and cell permeability improvement of lamivudine* via *COS nanosystems.* To increase the lipophilicity of a water-soluble antiviral drug, the prodrug of lamivudine (LA), lamivudine stearate (LAS), was synthesized via ester linkage between lamivudine and stearic acid. Stearic acid-g-CS oligosaccharide (CSO-SA) micelles demonstrated fast internalization and accumulation in tumor cells. The LAS-loaded CSO-SA micelles (CSO-SA/LAS) demonstrated high entrapment efficiency and drug loading. LA release from CSO-SA/LAS showed a pH-dependent behavior. In vitro, anti-HBV activities of CSO-SA/LAS presented more conspicuous inhibitory effects on antigen expression and DNA replication compared to LA and LAS. The CSO-SA/LAS micelle delivery system exhibited relatively low cytotoxicity, high uptake, and conspicuous in vitro anti-HBV activities, while the blank CSO-SA micelles possessed some antiviral activities, as well. Overall, the micelles are a promising carrier for effective therapy of anti-HBV drugs [253].

## 5. Conclusions

This review integrates several key aspects relevant to the development of progressive dosage forms and delivery systems for antivirals. Firstly, it provides a comprehensive view of currently used antivirals classified according to the mechanism of action in the organism. Based on this background, new findings concerning innovative dosage forms of 2nd and 3rd generations are discussed. Within these innovations, chitosan-based antivirals and antiviral delivery systems resp. carriers are highlighted. Chitosan and its derivatives are presented as highly potent polymeric as well as nanoparticulate systems accomplishing various important goals in modern antiviral therapy. 

It can be summarized there is great interest in developing new sophisticated antiviral systems due to the many limitations of current antiviral therapies and the growing viral pandemics. It is important to have effective means of preventing viral transmission and treating a viral infection in humans and also animals. Although many antivirals are already available, their efficacy is often limited because of factors such as poor solubility, low permeability, poor bioavailability, untargeted release, toxicity, adverse side effects, and antiviral resistance. The development of a new antiviral drug is time-consuming and expensive; therefore, the research is oriented toward innovative dosage forms of known antivirals. Many of their limitations can be overcome using advanced antiviral delivery systems constructed on biodegradable polymers (native as well as derivatized) and corresponding nanotechnology principles.

In this context, CS NPs are one of the most popular biopolymer-based NPs used for targeting the delivery of antivirals. They provide high drug encapsulation efficiency. They are often used to create controlled-release drug delivery systems (prolonged or sustained) capable of delivering the drug for longer, which maintains a stable level of the drug in the circulation, as well as reducing the dosing, side effects, and toxicity. CS NPs also have a synergic effect when combined with antiviral drugs and the necessary amount of drug can be reduced. This leads to reduced toxicity, which is another key reason for creating CS NPs. Pharmaceutical vehicles based on CS NPs can be capable of enhancing microbicide performance for better protection from sexually transmitted diseases. A very important reason to use CS NPs as an antiviral drug carrier is improved bioavailability. Oral bioavailability is increased because an antiviral drug encapsulated in CS NPs is protected against gastric degradation, and CS increases uptake by opening the tight junctions in the intestinal epithelium. CS NPs can increase permeation and thus improve the topical, nasal, or vaginal bioavailability of antivirals. Some CS derivates and combinations of CS with other materials can improve the solubility of low-soluble antiviral drugs, thus enhancing their bioavailability and reducing toxicity and dosing. Notable is how CS NPs consisting of specific CS derivates (such as carboxyalkylated, sulfated, quaternized, oligosaccharides) can possess antiviral activity themselves.

Although the use of commercial CS products in drug delivery (at a research or clinical level) could be limited, to some extent, by its structural variability among batches (depending on the CS source and its preparation), the benefits will undoubtedly accelerate its implementation in innovative dosage forms and carrier systems for antivirals, to achieve effective therapy for viral diseases.

## Figures and Tables

**Table 1:** Current antivirals divided according to their mechanism of action, drug administration approaches, and dosage forms.

Fusion (Attachment) Inhibitors						
Antiviral Drug	Mechanism of Action	Administration/Dosage Form	Diseases	Advantages	Limitations	References
Enfuvirtide 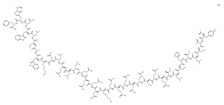	-interferes with the entry of HIV-1 into cells by inhibiting the fusion of viral and cellular membranes -binds to the first heptad-repeat (HR1) in the gp41 subunit of the viral envelope glycoprotein and prevents the conformational changes required for the fusion of viral and cellular membranes	Subcutaneous route/injection	AIDS	-increases the number of CD4 cells -reducing the amount of HIV in the blood reduces the risk of death or infections due to low immunity	-adverse effects: depression, nervousness, tiredness, muscle pain, nausea, loss of appetite, weight loss, diarrhea, constipation, flu-like symptoms, swollen glands, or painful, red, or teary eyes	[29,30]
**DNA Polymerase Inhibitors (DPIs)**						
Idoxuridine 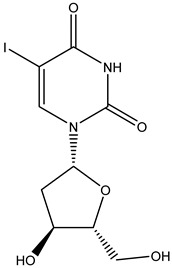	Nucleoside inhibitor -inhibits viral replication by substituting itself for thymidine in viral DNA. This, in turn, inhibits thymidylate phosphorylase and viral DNA polymerases from properly functioning. The effect of idoxuridine results in the inability of the virus to reproduce or infect/destroy tissue	Ocular route/ointment, solution	Feline herpetic keratitis and conjunctivitis	-potential anti-cancer effects thanks to its cytotoxicity	-cardiotoxicity, just for local use -burning, stinging, pain, irritation, itching, redness, blurred vision, eyelid itching, eyelid swelling, or sensitivity to light	[31,32]
Vidarabine 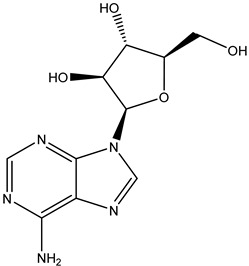	-stops replication of herpes viral DNA in two ways: (1) competitive inhibition of viral DNA polymerase, and consequently, (2) incorporation into and termination of the growing viral DNA chain	Ocular route/ointment	Herpes and acyclovir-resistant viruses Varicella zoster	-reduces lesion formation and the duration of viral shedding -less susceptible to the development of drug-resistant strains than other antivirals	-burning, stinging, pain, irritation, itching, redness, swelling, blurred vision, tearing, feeling like something is in the eye, or sensitivity to light	[33]
Acyclovir 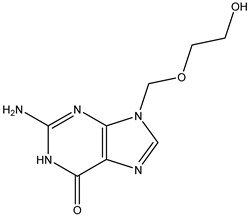	-acyclovir triphosphate competitively inhibits viral DNA polymerase by acting as an analog to deoxyguanosine triphosphate (dGTP) -incorporation of acyclovir triphosphate into DNA results in chain termination since the absence of a 3′ hydroxyl group prevents the attachment of additional nucleosides	Oral route/tablet Intravenous route/injection Transdermal route/ointment, cream	Herpes simplex virus infections Herpes zoster infection Varicella zoster virus infection Cytomegalovirus infection	-prevention of recurrent genital herpes infections -helps relieve the herpes pain and discomfort and helps the sores heal faster	-nausea, vomiting, burning, stinging, pruritus, rash, urticaria, headache, diarrhea, occasionally renal insufficiency and neurotoxicity -absorbed drug reaches the breast milk, placenta, and amniotic fluid	[34,35,36]
Cidofovir 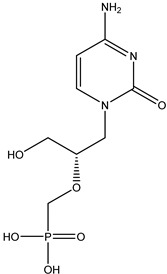	Nucleotide inhibitor -acts as a competitive inhibitor and an alternate substrate for cytomegalovirus (CMV) DNA polymerase	Intravenous route/infusion	Cytomegaloviral retinitis in people with AIDS	-used with probenecid to treat a certain viral eye infection -lowers the risk of blindness and other vision problems	-nausea, vomiting, diarrhea, loss of appetite, white patches or sores inside mouth or on lips, headache, skin rash, hair loss, or cough	[37]
Foscarnet 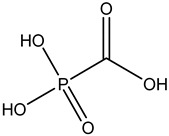	Pyrophosphate analog -interacts with the enzymatic action of polymerases and inhibits the cleavage of pyrophosphate from the nucleoside triphosphate -a non-competitive inhibitor of herpesvirus DNA polymerase, hepatitis B virus DNA polymerase, and reverse transcriptases	Intravenous route/infusion	Cytomegalovirus infection	-successful in the treatment of limited numbers of immunocompromised patients with CMV-associated gastrointestinal (improvement in over 67% of patients) and other infections	-mineral and electrolyte imbalances, neurotoxicity, nausea, vomiting, anemia, bone marrow suppression, decreased creatinine clearance, or conjunctivitis	[30,38]
Phosphonoacetic acid 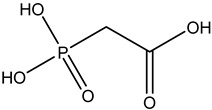		Intravenous route/infusion	Herpes simplex 1 infection Herpes simplex 2 infections Epstein–Barr virus infection Cytomegalovirus infection	-lack of toxicity toward many animal cells	-nausea, vomiting, anemia, bone marrow suppression, decreased creatinine clearance	[39,40]
**Reverse Transcriptase Inhibitors**						
**Zidovudine** 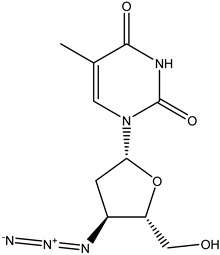	Nucleoside analog -active against HIV, a retrovirus. The drugs inhibit RNA virus replication by reversible inhibition of viral HIV reverse transcriptase, which reverse transcribes viral RNA into DNA for insertion into the host DNA sequence	Oral route/tablet Intravenous route/injection	AIDS	-will not cure or prevent HIV infection or AIDS; however, it helps keep HIV from reproducing and appears to slow down the destruction of the immune system	-monotherapy is recommended only in the initial management of HIV-1-infected patients -bone marrow suppression -combination therapy in advanced disease (zidovudine in combination with lamuvidine as combivir and with lamuvidine and abacavir as trizvir	[41]
Didanosine 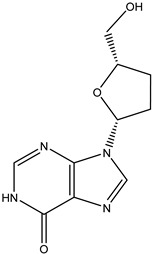		Oral route/capsule	AIDS	-helps to decrease the amount of HIV in the body so the immune system can work better. -this lowers the chance of getting HIV complications	-peripheral neuropathy -pancreatitis, lactic acidosis, hepatomegaly, hyperuricaemia -similar activity to zidovudine	[30,42,43]
Zalcitabine 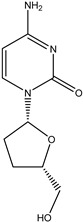		Oral route/tablet	AIDS	-blocking the growth of HIV -used in combination with other medicines	-peripheral neuropathy, nausea, vomiting, headache, hepatotoxicity, or cardiomyopathy	[44]
Stavudine 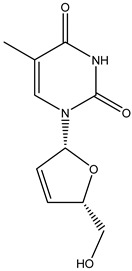		Oral route/capsule, solution	AIDS	-helps to decrease the amount of HIV in the body so your immune system can work better	-numbness, tingling, pain in hands or feet, weakness, liver problems, stomach pain, loss of appetite, dark urine, clay-colored stools, jaundice (yellowing of the skin or eyes), pancreatitis, fever, nausea, or vomiting	[45]
Lamivudine 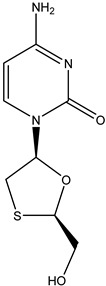		Oral route/tablet	AIDS Chronic hepatitis B	-lamivudine therapy is associated with a significant improvement in hepatic histology, normalization of hepatic enzymes, and suppression of plasma HBV DNA	-headache, nausea, fatigue, dizziness, neutropenia, or skin rash	[46,47]
Tenofovir 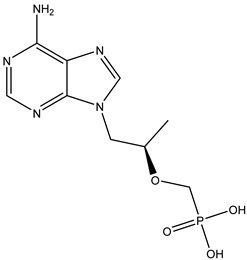	Nucleotide analog -inhibits HIV-1 reverse transcriptase and the hepatitis B polymerase through direct binding competition with the natural deoxyribonucleotide substrate (deoxyadenosine 5′-triphosphate) and, after integration into DNA, causes viral DNA chain termination	Oral route/tablet	AIDS Chronic hepatitis B infection	-in HBeAg-negative patients, tenofovir was the most effective in inducing undetectable levels of HBV DNA (94%) and improving liver histology (65%); it ranked second for normalization of ALT levels (73%)	-side effects: diarrhea, nausea, fatigue, headache, dizziness, depression, insomnia, abnormal dreams, and rash	[46,48]
Adefovir 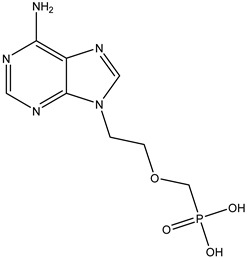		Oral route/tablet	Chronic hepatitis B infection	-provides sustained suppression of the virus and improvement in liver disease	-weakness, headache, fever, increased cough, nausea, vomiting, diarrhea -risk of lactic acidosis and hepatomegaly with steatosis -patients with renal dysfunction since chronic administration may result in nephrotoxicity	[49]
Efavirenz 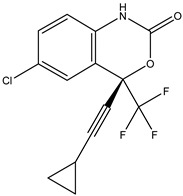	Nonnucleoside analog -directly inhibits the HIV-1 reverse transcriptase by binding in a reversible and non-competitive manner to the enzyme	Oral route/capsule, tablet	AIDS	-improves the function of the immune system by decreasing the amount of HIV in the body	-neuropsychiatric side effects: dizziness, headache, insomnia, impaired concentration, or abnormal dreams	[43,49]
Nevirapine 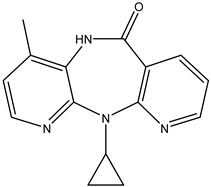		Oral route/tablet	AIDS	-prevents mother-to-child transmission of HIV-1 in pregnant women	-induces the metabolism of warfarin -side effects: nausea, headache, or rash	[45,50]
Delavirdine 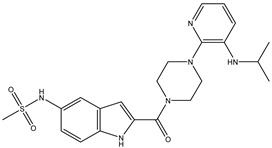		Oral route/tablet	AIDS	-is generally well tolerated -patients achieve marked improvements in virological and immunological markers -favorably increases plasma concentrations of several protease inhibitors, and the drug may also be beneficial as a component of salvage therapy in combination with protease inhibitors	-associated with a low rate of transient serum aminotransferase elevations during therapy and is a rare cause of clinically apparent acute liver injury -maculopapular rash	[30,51]
Etravirine 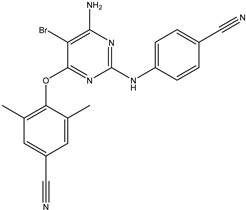		Oral route/tablet	AIDS	-does not need metabolic activation -decreases the amount of HIV in the blood	-side effects: rash, headache, nausea, diarrhea, fatigue, hypertension, abdominal pain, or peripheral neuropathy	[30]
**Integrase Inhibitors**						
Raltegravir 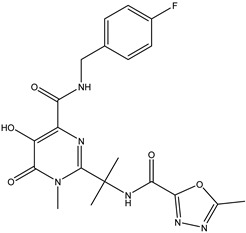	-stops integrase from working, which stops HIV from entering CD4 cells -the medication does not cure HIV, but it keeps the virus from multiplying -as part of an antiretroviral treatment plan, it helps reduce the amount of HIV in the body to undetectable levels	Oral route/tablet, chewable tablet, granules for oral suspension	AIDS	-lowers the chance of getting HIV complications -supports the immune system by decreasing the amount of HIV in the blood so it can work better	-metabolized by glucuronidation -adverse effects: nausea, diarrhea, or headache -myopathy and rhabdomyolysis are connected with the drug associated with muscle toxicity	[30,52]
**Portmanteau Inhibitors**						
Caffeoyl-anilide compounds 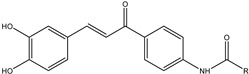	-function as a reverse transcriptase inhibitor as well as an integrase inhibitor	Intravenous route/injection	AIDS	-strategy to reduce the pill burden -dual action in inhibiting HIV integrase and blocking the CCR5 receptor-mediated entry	-price -high in toxicity because many drugs are taken at one time	[53]
**Protease Inhibitors**						
Saquinavir 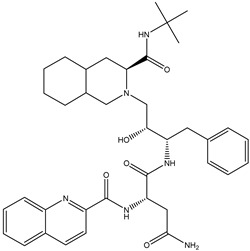	-inhibits the cleavage of the polyprotein into functional proteins -protease is a protein-based enzyme that normally breaks the polyprotein into functional proteins, so blocking or inhibiting protease prevents this essential step of viral reproduction	Oral route/capsule, tablet	AIDS	-in combination with ritonavir and other HIV medications, is used to help control HIV infection -it helps to decrease the amount of HIV in the body so the immune system can work better -only four percent of patients receiving saquinavir had side effects	-potential side effects: insulin resistance, nausea, diarrhea, development of gallstones or kidney stones, changes in how things taste, insomnia, elevated numbers in liver function tests, rash or dry skin, or elevated cholesterol	[54,55]
Indinavir 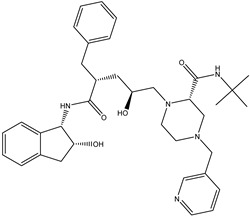		Oral route/capsule	AIDS	-recent studies have analyzed the effects of indinavir as an anti-cancer agent (human papillomavirus is induced by increased expression of eukaryotic translation initiation factor 4E (eIF4E), potentially leading to cervical cancer)	-side effects: nausea, vomiting, fatigue, diarrhea, kidney stones, nephrolithiasis (for solubility only in acidic conditions), or kidney inflammation	[56]
Amprenavir 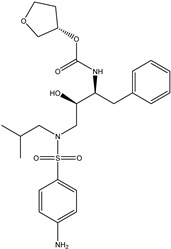		Oral route/capsule	AIDS	-greatly increased water solubility and improved oral bioavailability -this allows a reduction in the daily dose	-general adverse effects: nausea, vomiting, diarrhea, epigastric pain, flatulence, paresthesia, headache, rash, or fatigue	[57,58]
Nelfinavir 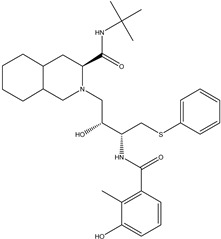		Oral route/tablet, powder	AIDS	-has also shown anti-cancer effects in in vitro and in vivo studies -supports the immune system with a lower amount of HIV in the blood	-low white blood cell counts, nausea, diarrhea, gas, stomach pain, loss of appetite, rash, or changes in the shape or location of body fat	[59,60]
Ritonavir 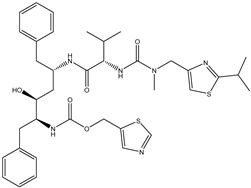		Oral route/tablet, soft gel capsule, oral suspension	AIDS SARS-CoV-2 infection	-The COVID-19 Treatment Guidelines Panel recommends against the use of lopinavir/ritonavir and other HIV protease inhibitors for the treatment of COVID-19 in hospitalized and non-hospitalized patients	-general side effects: drowsiness, diarrhea, gas, heartburn, change in the ability to taste food, headache, numbness, burning, tingling of the hands, feet, or area around the mouth, muscle or joint pain, stomach pain, nausea, or vomiting	[61]
**Signaling Inhibitors**						
Ribavirin 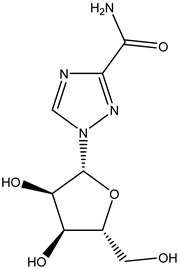	-directly inhibits viral mRNA polymerase by binding to the nucleotide-binding site of the enzyme -prevents the binding of the correct nucleotides, leading to a reduction in viral replication or the production of defective virions -administered as a combination therapy -inhibits inosine monophosphate dehydrogenase	Oral route/tablet Intravenous route/injection Inhalation route/aerosol	SARS-CoV-2 infection Hepatitis C Severe respiratory syncytial virus infection	-improves the signs and symptoms of viral bronchiolitis in infancy	-not effective when used alone; the potential is tripled when in combination with lopinavir/ritonavir and interferon-beta-1b, which alleviated symptoms of SARS-CoV-2 completely within four days -common side effects: cough, upset stomach, vomiting, diarrhea, constipation, heartburn, loss of appetite, or weight loss	[62,63]
Viramidine 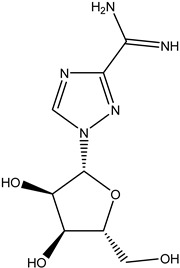		Oral route/tablet	Chronic hepatitis C	-viramidine is taken up into hepatocytes by a mechanism distinct from that of ribavirin, with greater affinity for the liver	-side effects: hemolytic anemia, vomiting, diarrhea, or loss of appetite	[64,65]

**Table 2:** CS derivatives applied as antivirals or antiviral carriers, with properties and preparations.

CS Derivatives	Formula	Physical Properties	Biological Properties	Preparation Method	Advantages/Limitations/Potential Uses	References
**Ionic Derivatives**						
Quarternized derivatives	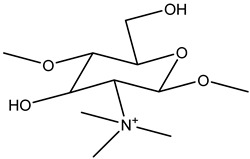	-cationic derivatives -water-soluble at neutral pH -N,N, N-trimethyl CS chloride (TMC) ↑ aqueous solubility than CS	-biocompatibility -biodegradability -mucoadhesion	-direct quaternary ammonium substitution -epoxy derivative open loop -N-alkylation	-antifungal, antibacterial, antituberculosis -enzyme inhibition -permeation enhancers -gene transfection and delivery -good moisture retention and absorption -mucoadhesivity ↓ with ↑ degree of quaternization -↑ degree of quaternization ↓ intrinsic viscosity -pH 7.4 CS and salts failed to increase the permeability -absorption enhancer for intestinal lumen with pH close to its pKa -TMC collects and delivers more negatively charged DNA/genes than plain CS -quaternized CS ↑ hydroxyl radical scavenging activity in comparison to other CS -pH-sensitive targeting	[184]
Sulfated derivatives	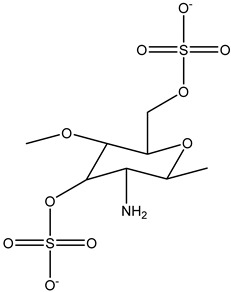	-water-soluble sulfoethylated CS with improved swelling property -during sulfation, some amino groups of CS are converted to anionic centers, which leads to better polyelectrolyte properties -N-alkyl-O-sulfated CS has amphiphilicity since it carries long-chain alkyl groups with a hydrophobic nature and sulfated groups with a hydrophilic nature	-biocompatibility -biodegradability	-sulfation of 2-chloroethane sulfonic acid sodium salt in alkaline media -sulfur-containing derivatives were obtained by reacting CS with CS2, formaldehyde, and primary amine	-antisclerotic, antioxidant, antibacterial, anti-HIV, antiviral, and enzyme inhibition -blood anticoagulant -hemagglutination inhibition activity -amphiphilic polymer enables the formation of micelles with physical entrapment of water-insoluble drugs such as taxol -structural similarity of CS salt with heparin -high sorption capacities -a great advantage for metal ion recovery	[25]
CS derivatives with sugar part	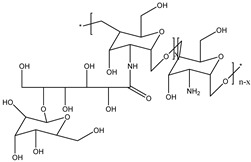	-water-soluble -investigated mainly for rheological studies	-biodegradability -biocompatibility -nontoxic	-reductive N-alkylation (using NaCNBH_3_ and unmodified sugar, a sugar-aldehyde derivative, or N-alkylation of CS performed in aqueous methanol with various aldehydes, monosaccharides, and disaccharides)	-antibacterial and antimicrobial activity -this type of modification has usually been used to introduce cell-specific sugars into CS -synthesis of sugar-bound CS, such as those with D- and L-fucose, and their specific interactions with lectin and cells -lactose-modified CS for a potential application in the repair of the articular cartilage -galactosylated CS prepared from lactobionic acid and CS with 1-ethyl-3-(3-dimethyl aminopropyl)- carbodiimide (EDC) and N-hydroxysuccinimide (NHS) showed promise as a synthetic extracellular matrix for hepatocyte attachment -graft copolymers of galactosylated CS with poly(ethylene glycol) or poly(vinyl pyrrolidone) were useful as hepatocyte-targeting DNA carriers	[181,185,186]
CS glutaraldehyde crosslinked polymer	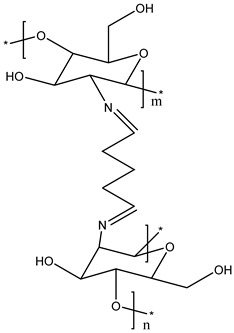	-improved permeability, mechanical properties, wetting, and chemical resistance -gelation temperature: 32–33 °C -viscosity of the hydrogel increased quickly after gelation	-not cytotoxic to human corneal epithelial cells at a low concentration -biocompatibility -biodegradability	-CS dissolved in acetic acid with glutaraldehyde performed in a short time	-N-trimethylated CS crosslinked with glutaraldehyde has been used to fabricate hollow microspheres for drug loading	[187]
CS cyclodextrin derivatives	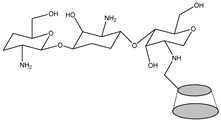	-can enhance the solubility of sulfadiazine, sulfamonomethoxine, and sulfamethoxazole	-antimicrobial activity -antifungal activity -bioavailability -nontoxic -biodegradability	-physical mixing, kneading, co-precipitation, and solvent evaporation	-increases dissolution -improves stability	[188,189,190]
CS oligomers	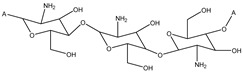	-soluble over a wide pH range, from acidic to basic -water-soluble	-antioxidant activity -anti-inflammatory activity -antiviral activity -nontoxic -antifungal activity -biodegradability -biocompatibility -immunological and antibacterial activities	-prepared from the degradation of CS -oxidative degradation method involving hydrogen peroxide (H_2_O_2_) and combined degradation using hydrogen peroxide and microwave radiation	-inhibit the expression of TNF-α, IL-6, and iNOS, which are associated with an inflammatory response -depress oxidative stress -decrease induced cell apoptosis -alleviate or delay Alzheimer´s disease process	[191,192]

↑ means increase, ↓ means decrease.

**Table 3:** CS NPs in drug delivery systems of antivirals.

Drug Delivery System	Virus	Mechanism of Action	Limitations and Advantages	State of Implementation In Practice	References
CS NPs (zinc)	HIV-1	-decrease the viral load -mostly interfere with the active replication of the virus, thereby decreasing its copy number	-greater effectiveness on resistant strains of microbial pathogens -less toxicity and heat resistance -biocompatibility and biocidal effect	-research study	[245,246]
CS NPs (silver)	H1N1 influenza A	-binding to the negatively charged protein capsid with subsequent destabilization of the envelope and altered permeability -inhibit the viral contact with host cells and interaction of silver NPs with viral glycoproteins	-no adverse effect of L-B-L coating on the physical properties of the fabric -sustained release of Ag+ from CS-Ag-NP-coated fabric -antibacterial, antifungal, anti-inflammatory, antiviral, anti-angiogenesis, and antiplatelet activities -exhibit a higher rate of cell destruction than common agents used as reference -good biocompatibility and dose-dependant toxicity	-research study	[247]
Polyquarternary phosphonium oligo CS NPs (nanosilver)	Hepatitis A	-induce ribonuclease catalyzed by CS to degrade the viral RNA and consequently prevent its transcription and translation	-NBCs are more virucidally active than PQPOCs against the target virus -the antiviral activity of NBCs is concentration- and pH-dependent	-research study	[248]
HTTC (*N*-(2-hydroxypropyl)-3-trimethylammonium CS) NPs	SARS-CoV-2	-exhibit effective inhibition of SARS-CoV-2 -complex formation between HTCC polymer and S-protein of the virus, which blocks viral entry into the host cell	-efficiently hamper infection of all low-pathogenicity human coronaviruses in vitro and ex vivo -replication of both viruses (SARS-CoV-2 and MERS-CoV) is efficiently hampered	-research study	[249]
GCPQ (*N*-palmitoyl-*N*-monomethyl-*N*,*N*-dimethyl-*N*,*N*,*N*-trimethyl-6-*O*-glycolCS)	SARS-CoV-2	-bind electrostatically to coronavirus S proteins to block not only entry to host cells but also viral replication	-mucoadhesive and chemically stable -limit viral cell entry in the nasal cavity, which could have a profound impact on the course and severity of the disease	-research study	[250]
CS-arginine derivative NPs	H1N1 influenza A, herpes simplex-1, encephalomyocarditis virus	-increase permeability of virus protein capsid	-good swelling degree, improved hydrophilicity, and biocompatibility in terms of surface-free energy components, which supports their application for tissue regeneration	-research study	[28,251]
CS NPs (IFNα-2b)	Human lymphotropic-T virus type 1 Vesicular stomatitis virus	-regulate the expression of many genes involved in the suppression of cell proliferation via Janus kinase/signal transducer and activator of transcription signaling pathway and the inhibition of viral replication	-ionotropic gelation method for the production of the nanodrug delivery system -water-based, simple, reproducible, cost-viable, and eventually scalable in an industrial setting -enhance oral absorption	-preliminary pharmacokinetic study	[252]
CS NPs (acyclovir)	HSV-1, HSV-2, CMV	-competitively inhibit viral DNA polymerase by acting as an analog to deoxyguanosine triphosphate (dGTP)	-use crosslinked CS with tripolyphosphate (TPP)	-research study	[19]
CS NPs (saquinavir)	HIV	-control viral proliferation as measured using two viral strains, NL4-3 and Indie-C1, and two target T-cells, Jurkat and CEM-CCR5 -bind to the active site of the viral protease and prevent cleavage of viral polyproteins, thus preventing maturation of the virus	-biopolymer-based NPs -ionic gelation technique -superior drug-loading potential with greater cell-targeting efficiency, leading to efficient control of the viral proliferation in target T-cells	-research study	[19]
CS-g-HPβCD NPs (efavirenz)	HIV	-non-nucleoside reverse transcriptase (RT) inhibitor of human immunodeficiency virus type 1 (HIV-1) -non-competitive inhibition of HIV-1 RT	-NPs showed sustained efavirenz release (99.03 ± 0.30% in 8 h) and followed the Fickian diffusion mechanism -NPs showed 4.76 times greater permeability than un-encapsulated efavirenz solution through the porcine nasal mucosa -development of intranasal mucoadhesive EFV-NPs for CNS targeting	-research study	[19]
Lamivudinestearate (LAS) loaded to stearic acid-g-CSoligosaccharide (CSO-SA) micelles (CSO-SA/LAS)	HBV	-must be converted intracellularly to its triphosphate form, which then competes with cytosine triphosphate for incorporation into the developing viral DNA strand	-faster release behavior of LA from CSO-SA micelles at low pH presents the potential for efficient antiviral agent delivery -exhibit relatively low cytotoxicity, high uptake, and conspicuous in vitro anti-HBV activities, while the blank CSO-SA micelles conduct some antiviral activities, as well -promising carriers for effective therapy of anti-HBV drugs	-research study	[253]
CS nanodroplets (sulfobutyl ether-β-cyclo-dextrin inclused with acyclovir)	HSV-2	-competitively inhibit viral DNA polymerase by acting as an analog to deoxyguanosine triphosphate (dGTP)	-ACV loaded into nanodroplets shows higher antiviral activity against HSV-2 in cell cultures compared to the free drug -higher intracellular concentration of the drug delivered by the nanodroplets -drug was complexed with sulfobutyl ether-β-cyclo-dextrin and then incorporated in the nanodroplet CS shell via electrostatic interaction	-in vitro study	[238]
Curdlan sulfate/CS polyelectrolyte complexes (CRDS/CS PECs) NPs (zidovudine)	HIV	-inhibit the HIV reverse transcriptase enzyme competitively and act as a chain terminator of DNA synthesis	-fabricated CRDS/CSPECs could be explored as potential nanocarriers for delivering antiviral drugs -cytotoxicity and antiviral activity in vitro of the AZT-loaded PECs are currently being studied	-in vitro study	[254]
GO/SPION/CS (graphene oxide/superparamagnetic iron oxide NPs/CS)	SARS-CoV-2	-GO could directly interact with viruses via electrostatic interactions, hydrogen bonding, and redox reactions -GO can absorb charged lipids and destroy membranes, suggesting the possibility of interaction with enveloped viruses such as the SARS-CoV-2 virus -inactivate viruses by attaching to the tail fiber and dissociating a virus, interfering with the viral replication process, binding to the viral ligands, and preventing their interaction with the host cell receptors	-capable of neutralizing the SARS-CoV-2 virus effectively and could be considered a promising material against COVID-19 -with decreasing sample magnetization, the relative magnetic cytotoxicity percentage shows a downward trend -no sign of antiviral activity is observed for SPION NPs -GO/SPION reveals merely 23% viral inhibition -the highest level of inhibition is for GO/SPION@CS, with more than 86% viral inhibition	-in vitro study	[255]
CS NPs (foscarnet)	CMV, HIV	-block the pyrophosphate binding site, preventing cleavage of pyrophosphate from deoxynucleotide triphosphates	-NPs showed no toxicity on non-infected HELF cells -crosslinked NPs showed controlled drug release	-in vitro study	[256]
CS NPs (sylimarine)	SARS-CoV-2	blocki viral host receptor ACE2, thus preventing viral attachment and its entry into cells	-take the form of intranasal drug delivery to inhibit viral entry -mucoadhesive property healing in its application as intranasal vaccine targeting mucosal pathway inducing local humoral and cellular immune responses	-in silico and in vitro study	[257]
CS/PEO (polyethylene oxide) nanofibrous mat (tenofovir)	HSV-2	-reduces TDF uptake and saturation of the enzymes involved in prodrug hydrolysis	-uniform drug distribution -mucoadhesive and swelling activity -better retention time with low leakage from the vaginal cavity -reduced TDF uptake and saturation of the enzymes involved in prodrug hydrolysis -fluctuations in the physiological pH range of 3.8–5.0 substantially affect mucoadhesive behavior and drug dissolution rate from the nanofibrous carrier -more acidic vaginal environment favors nanofibrous mat adherence to the human vaginal tissue and speeds up drug diffusion from the polymer matrix, which, in turn, may increase the drug residence time with mucosal tissue and help to initiate microbicide absorption onset	-in vitro studies	[258]
CS-SeNPs	PRRSV (porcine reproductive and respiratory syndrome virus)	-suppress oxidative stress induced by rPRRSV-EGFP infection by increasing GSH-Px activity, promoting GSH production, and inhibiting H_2_O_2_ synthesis -enhance the antioxidant capacity and effectively suppress PRRSV-induced apoptosis in Marc-145 cells via the ROS/JNK signaling pathway, thereby inhibiting PRRSV replication	-inhibit ORF5 gene expression, viral titers, and N protein of r-PRRSV-EGFP at 24 and 48 h post-infection (hpi) in Marc-145 cells	-in vitro study	[259]
CS-based polymeric NPs (dolutegravir)	HIV	-inhibit HIV integrase by binding to the active site and blocking the strand transfer step of retroviral DNA integration in the host cell	-no toxicity associated with the administration of the NPs through spray-drying technology as a milk admixture for pediatrics -organ biodistribution of the drug was significantly higher when administered as a nanoformulation than the pure drug -coadministration of the drug along with milk does not affect the absorption coefficient in vivo; the time taken to reach the maximum concentration is delayed	-in vivo study using Balb-C mice models	[260]
CS NPs (nevirapine)	HIV	-bind directly to reverse transcriptase and blocks the RNA-dependent and DNA-dependent DNA polymerase activities by disrupting the enzyme’s catalytic site	-very good permeation of the drug across the vaginal tissue -increase the bioavailability in the target area -increase the therapeutic effect -reduce the systemic toxicity -pharmacokinetics and pharmacodynamic study and safety and efficacy assessments are not finished	-ex vivo study	[261]

## Data Availability

Data are available from the authors.
